# A CO_2_ electrolyzer tandem cell system for CO_2_-CO co-feed valorization in a Ni-N-C/Cu-catalyzed reaction cascade

**DOI:** 10.1038/s41467-023-41278-7

**Published:** 2023-09-14

**Authors:** Tim Möller, Michael Filippi, Sven Brückner, Wen Ju, Peter Strasser

**Affiliations:** The Electrochemical Energy, Catalysis, and Materials Science Laboratory, Department of Chemistry, Chemical Engineering Division, Technical University Berlin, Berlin, Germany

**Keywords:** Electrocatalysis, Energy storage, Electrocatalysis, Chemical engineering

## Abstract

Coupled tandem electrolyzer concepts have been predicted to offer kinetic benefits to sluggish catalytic reactions thanks to their flexibility of reaction environments in each cell. Here we design, assemble, test, and analyze the first complete low-temperature, neutral-pH, cathode precious metal-free tandem CO_2_ electrolyzer cell chain. The tandem system couples an Ag-free CO_2_-to-CO_2_/CO electrolyzer (cell-1) to a CO_2_/CO-to-C_2+_ product electrolyzer (cell-2). Cell-1 and cell-2 incorporate selective Ni-N-C-based and Cu-based Gas Diffusion Cathodes, respectively, and operate at sustainable neutral pH conditions. Using our tandem cell system, we report strongly enhanced rates for the production of ethylene (by 50%) and alcohols (by 100%) and a sharply increased C_2+_ energy efficiency (by 100%) at current densities of up to 700 mA cm^−2^ compared to the single CO_2_-to-C_2+_ electrolyzer cell system approach. This study demonstrates that coupled tandem electrolyzer cell systems can offer kinetic and practical energetic benefits over single-cell designs for the production of value-added C_2+_ chemicals and fuels directly from CO_2_ feeds without intermediate separation or purification.

## Abstract (web summary)

Tandem concepts for electroreduction of CO_2_ offer a valuable toolkit to tackle sluggish reaction kinetics and raise the production of e-chemicals. Here, the authors report a cascade system with two coupled electrolyzers using Ni-N-C and Cu-based catalysts for enhanced CO_2_ to multi-carbon conversion

## Introduction

In the light of intensified efforts to mitigate power and chemicals production from fossil sources and their substitution by renewable ones, electrocatalytic power-to-chemicals and power-to-fuels technologies are emerging as one of the future pillars of sustainable chemicals industry^1^. Recent years have seen a drastic increase in attention for the electrocatalytic CO_2_ reduction reaction (CO_2_RR), advancing the production of 2e^−^ reduction products, such as CO and formic acid_,_ towards commercialization^2–4^. In contrast, the electrocatalytic reaction process pathway towards C_2+_ hydrocarbons and oxygenates, in particular C_2_H_4_, Ethanol, Propanol, lacks similar technological advances^5–10^.

Following Hori’s pivotal work on CO_2_RR, establishing CO as key intermediate in hydrocarbons and oxygenates production, studies investigated CO as feedstock in what is referred to as the CO reduction reaction (CORR). CORR achieves a higher faradaic efficiency (FE) for the production of C_2+_ compounds, in part because some common C_1_ side-products of the CO_2_RR, such as CO or HCOO^-^, cannot be formed, but also due to more favorable kinetics and surface coverages of H^*^ and CO^*^. Specifically, the production of oxygenates has been shown to be favored by CORR compared to CO_2_RR, especially under alkaline conditions that are only sustainable for CORR due to the invariable formation of (bi)carbonate from CO_2_^11–19^.

The favorable kinetics of alkaline CORR compared to CO_2_RR for production of C_2+_ species has motivated researchers to try to find ways to separate off the CORR in a CO_2_RR electrolyzer. Tandem Cathode Concepts follow the idea to spatially decouple the initial 2e^-^ reduction of CO_2_ to CO and the subsequent reduction steps of CO towards C_2+_ products. The tandem split can be realized at vastly different length scales and distinct configurations: (i) at the atomic scale by two neighboring active sites coupled by CO spillover, (ii) at the macroscopic electrode scale with macroscopically separated regions of distinct catalysts inside the same electrolyzer cell, and (iii) at the macroscopic electrolyzer cell scale forming a coupled process cascade^20–35^. While the idea of placing the active sites of CO source and sink inside the same electrode is an intriguing one, it has been shown to be inherently sensitive to the relative spatial catalyst distribution that is challenging to maintain over longer operation times considering the reported mobility of Cu catalysts under CO_2_RR^36–41^.

The present work sets out to explore the coupled tandem electrolyzer cell approach, i.e., two individual electrolyzers in series, for a significantly enhanced production of C_2+_ compounds from CO_2_ in a catalytic reaction cascade. We deliberately avoid the use of high-temperature technologies, harsh reaction conditions, and demonstrate the advantages by avoiding CO_2_ scrubbing steps to increase the practicality of the overall process, and make use of kinetic benefits for CO_2_/CO co-feeds agreeing with the presence of reactant- and product-specific reaction sites on Cu catalysts^20,42^. Accordingly, we deploy a noble metal-free single Ni atom site electrocatalyst (Ni-N-C) in a neutral-pH, near-ambient temperature, zero-gap setup for selective conversion of CO_2_ to CO-rich CO_2_/CO mixed streams. The CO_2_/CO mixed feed was then directly introduced to a second flow electrolyzer cell equipped with a Cu-catalyst for conversion of CO_2_/CO mixed feeds into C_2+_ products. We report strongly enhanced rates for the production of ethylene (~50%) and of alcohols (>= 100%) and a sharply increased C_2+_ energy efficiency (>=100%) in the tandem systems compared to the conventional single CO_2_ electrolyzer cell approach. To validate our conclusions and provide kinetic insight, we also conducted single-cell control experiments with controlled mixed CO_2_/CO co-feeds to explore the origin of the observed tandem effect. These experiments suggest that CO_2_ is being selectively scrubbed from the gas mixture by cathodically generated OH^-^ in vicinity of the cathode thus locally increasing the effective CO concentration and CO surface coverage, mimicking the performance of CO-rich gas feeds.

## Results and Discussion

### Design of a low-temperature tandem electrolyzer cascade for the production of C_2+_ compounds

**Fig. 1:**
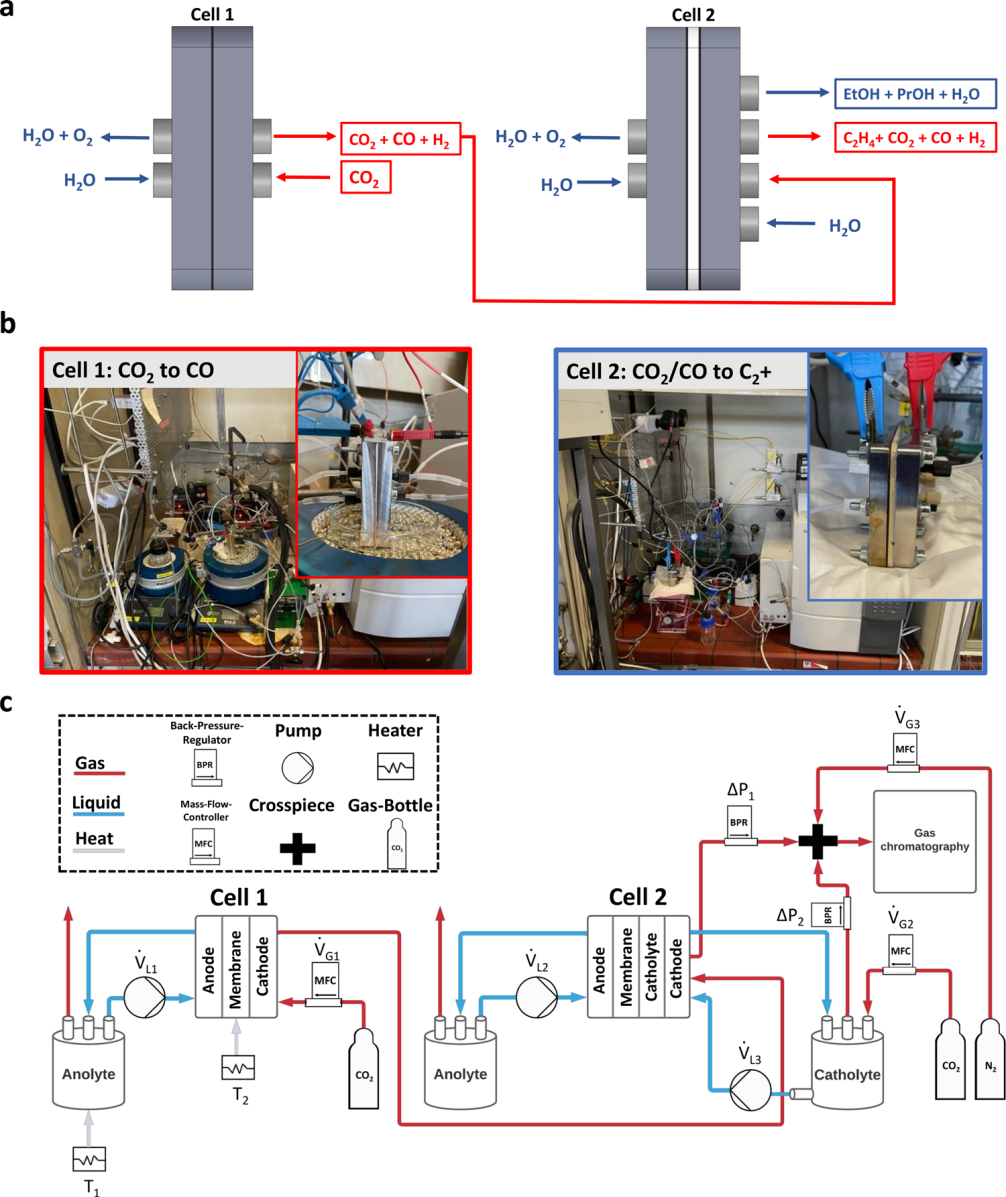
Illustration of the tandem electrolyzer cell system used for efficient CO_2_ valorization. **a** Concept of coupled Tandem CO_2_ Electrolyzer Cells highlighting the flows of gas (red) and liquid (blue) reactants and products. Additional information on the detailed reaction conditions can be found in Supplementary Fig. 2. **b** Photographs of the two electrolysis reactors used for coupled CO_2_-to-CO electrolysis and CO_2_/CO-to-C_2+_ electrolysis, with inserts showing an enlargement of the cells used in each case. **c** Detailed Process scheme of tandem cell set up with information on gas (red) and liquid (blue) flow directions and the most important components of the investigated tandem system.

Figure 1a introduces the concept and the process design of the first low-temperature coupled tandem CO_2_ electrolyzer cell system developed and studied in this contribution.

The numbering of the cells refers to the order in which they were deployed in the tandem system electrolyzer cascade and is used as a denomination throughout this work. Cell-1, the first electrolyzer in the reaction cascade, converts CO_2_ to CO and is the point of entry for the CO_2_ feed. At the cathode Gas Diffusion Electrode (GDE) of cell-1, we use a previously developed noble metal-free single Ni atom site electrocatalyst (NiNC) deployed into a neutral anode-pH, zero-cathode-gap electrode design^43^. Since the stoichiometric CO_2_ ratio, λ_stoich_, remains above one, the cathodic CO_2_ to CO conversion in cell-1 invariably results in mixed CO_2_/CO exit feeds, even if the cathode is operated at 100% faradaic CO efficiency. This mixed CO_2_/CO gas feed is directly used as input feed for a Cu-based electrolyzer “cell-2” to efficiently produce C_2+_ products, as schematically depicted in Fig. 1a. Note, that while within this work we refer to the commercial Cu catalysts as “Cu” for simplicity, XRD analysis showed that the material is partially oxidized and shows a presence of a Cu_2_O phase, see Supplementary Fig. 1.

Figure 1b shows photographs of our experimental setup with inserts of the individual electrolysis cells used during electrochemical characterization.

The detailed tandem cell process scheme is given in Fig. 1c. Note how cell-1 and cell-2 were connected in series by the CO_2_ gas flow to enable an enrichment with CO in the gas stream before entering cell-2. A more detailed discussion on the geometry of the individual cells and testing setup including operational parameters can be found in Supplementary Fig. 2.

### Electrochemical CO_2_/CO mixed gas feed reduction

Prior to coupled tandem electrolyzer cell experiments, we studied how the presence of CO in the CO_2_ reactant gas feed affects the product production rates of a single Cu cathode-based CO_2_ electrolyzer.

**Fig. 2:**
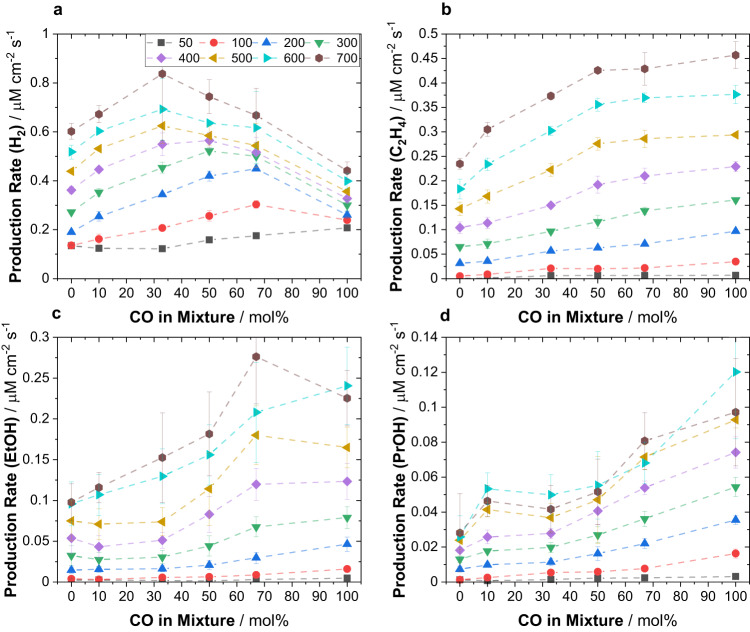
Influence of CO_2_/CO co-feed composition on production rates observed for electrolysis in single-cell experiments of cell-2. Production rates of key products using a single electrolyzer cell as a function of CO mol per cent (mol%) in the CO_2_/CO co-feed, measured for various applied current densities in Cu-based cell-2. Production rates are shown for **a** hydrogen, **b** ethylene, **c** ethanol and **d** n-propanol. Electrolysis was conducted in single-cell experiments deploying only the Cu-based electrolyzer denoted as cell-2. The total volumetric flow of co-feed mixtures was kept constant at 50 mL min^−1^ throughout all experiments, while the CO concentration was gradually increased. Displayed values represent the average and error bars the standard deviation of at least 2 independent measurements.

Figure 2 displays the production rates of the four most prominent reaction products, i.e., hydrogen, ethylene, ethanol, and n-propanol as a function of CO mol% in the CO_2_/CO co-feed and of applied current density. While the absolute volumetric flow of the gas feed was kept constant at 50 mL min^-1^, we have varied its composition by adjusting the individual flow rates of CO_2_ and CO that were mixed prior to entering cell-2. Figure 2a shows a profound effect of CO in the co-feed on the production of hydrogen. For CO-lean feeds, an increase in CO raised H_2_ production, as well, before H_2_ production rates dropped for CO-rich co-feeds. The molar CO concentration associated with the maximum H_2_ production depended on the applied current density. While for low current densities such as 100 or 200 mA cm^-2^ the turning point was at 66 mol%_CO_, larger current densities showed the transition at 50 mol%_CO_ (300 and 400 mA cm^-2^), or even at 34 mol%_CO_ (500 to 700 mA cm^-2^). The single-maximum pattern of hydrogen production appears surprising, as the total carbon content in the electrolyte monotonically drops with more CO due to the vastly lower solubility of CO versus CO_2_. So, CO_x_ surface coverages should monotonically decline, as well. For the production rate of ethylene, shown in Fig. 2b, a distinctly different trend was apparent. Here, increasing CO mol% improved the ethylene production rate. Note that this improvement was most significant up to a co-feed composition of 50 mol%_CO_, beyond which it plateaued. The production rates of ethanol and n-propanol, Fig. 2c, d, showed unusually sharp enhancements with rising molar CO mol% in the co-feed. In contrast to the trends observed for ethylene, the beneficial effect on production rate was most pronounced past 50 mol%_CO_ approaching pure CO gas feed. Production rates of other compounds such as formate, acetate and ally alcohol can be found in Supplementary Fig. 3. The trend for allyl alcohol generally agrees with the behavior observed for other alcohols, whereas formate production was suppressed by raising CO content in line with expectations.

To better appreciate the effect of CO in the feed, let us consider the kinetic enhancements of individual C_2+_ production rates on a relative scale to pure CO_2_ feeds: CO in the feed has the most significant effect on PrOH production, showing a 4x increase in rate, followed by a 2-3x increase of EtOH production and finally 2x increase in C_2_H_4_ production. This sharp enhancement of C_2+_ oxygenate production under CO-rich feed conditions is also reflected by the dramatically larger FE values obtained for pure CO feed (CORR) compared to pure CO_2_ feed (CO_2_RR), Supplementary Fig. 4. These enhancements can be explained mechanistically by enhanced cross-coupling CO dimerization rates on the surface of the Cu catalyst^20^. The combined presence of CO_2_ and CO gas in the feed offers new C-C coupling pathways between two adsorbed CO surface species derived from both CO_2_ and CO on two distinct, non-scrambling Cu surface sites, in line with the multi-site hypothesis on Cu catalysts^20,42^.

### Kinetic analysis of CO_2_RR and CORR of CO_2_/CO feeds

**Fig. 3:**
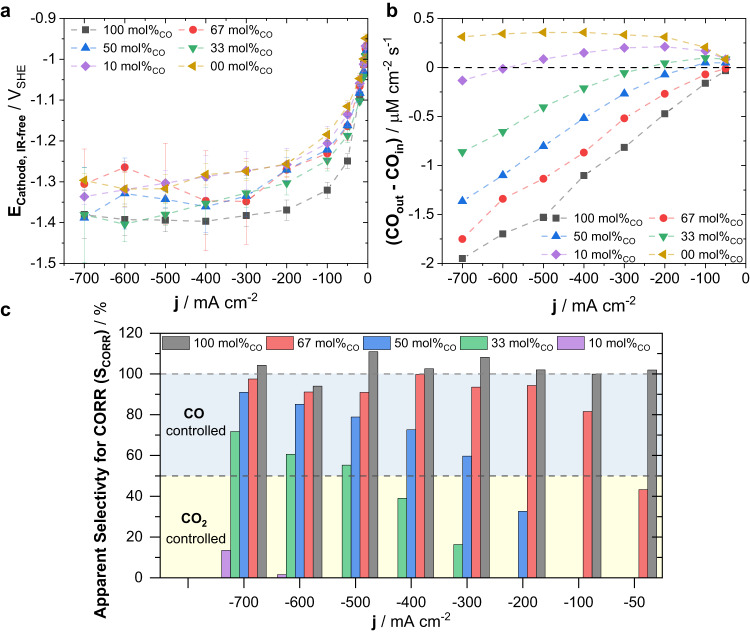
Electrode potential and CO conversion under CO_2_/CO co-feed conditions in single-cell experiments of cell-2. **a** IR-corrected cathode polarization curves as a function of applied current density for various investigated co-feed compositions. **b** The net CO flux, CO_net flux_, defined as the difference in the molar CO influx, CO_in_, and molar CO outflux, CO_out_, plotted versus the applied current density and co-feed composition. **c** Apparent selectivity for CORR, denoted S_CORR_, as a function of applied current density and co-feed composition. S_CORR_ was defined as the ratio between the electrocatalytic CO consumption rate (CO_consumption_ = –CO_net flux_) and the theoretically required molar CO consumption rate to produce all C_2+_ species by CORR. Colored areas indicate regions controlled by the respective primary reactant used in the production of C_2+_ species, either CO or CO_2_, dependent on the absolute value of S_CORR_. Electrolysis was conducted in single-cell experiments deploying only the Cu-based electrolyzer denoted as cell-2. The total volumetric flow of co-feed mixtures was kept constant at 50 mL min^−1^ throughout all experiments, while the CO concentration was gradually increased. Displayed values represent the average and error bars the standard deviation of at least 2 independent measurements.

To gain a better understanding of the origin of the CO dependence of the production rates, we tracked the applied cathode potentials as well as the CO consumption during co-feed experiments. Figure 3a shows IR-free cathode polarization curves obtained for experiments at varying CO feed concentrations. Evidently, cathode overpotentials (E_cathode, IR free_) during electrolysis in pure CO_2_ and in small molar CO co-feeds of 10 mol% remained near −1.3 V_SHE_ at a current density approaching 700 mA cm^–2^. In contrast, the required cathode potentials shifted to –1.4 V_SHE_ for the case of a pure CO feed, possibly pointing to concentration overpotentials due to lower CO solubility.

As our co-feeding experiments introduced two distinct reactants, i.e., CO_2_ and CO, we set out to quantify whether and how the shift in C_2+_ production rates with CO mol% correlated with a shift from predominantly CO_2_-controlled electroreduction to a predominantly CO-controlled one. For that purpose, we measured the CO outflux (area-normalized molar outflow rates) out of the electrolyzer at various co-feed compositions, Supplementary Fig. 5. Considering the large stoichiometric excess of reactants in the input feed, CO conversion was incomplete for all investigated co-feeds. Higher CO mol% in the feed led to higher molar CO outflux, CO_out_, from the electrolyzer. However, the decrease in CO_out_ with larger current density was significantly more pronounced for CO-rich co-feeds, suggesting a larger relative consumption thereof. Taking the difference between the molar CO influx, CO_in_, and the molar CO outflux during electrolysis, we derived the *net CO flux*, defined as CO_net flux_ = (CO_out_ - CO_in_), as function of applied current and co-feed composition, Fig. 3b. In this plot, positive and negative values of CO_net flux_ denote a catalytic net production and net consumption of CO, respectively. Generally, both higher CO mol% in the feed and larger current density led to increasing CO consumption, i.e., more negative values of CO_net flux_. For CO mol% <= 50 in the feed, there appeared a critical current density, beyond which net CO production turned into net CO consumption (10, 33 and 50 mol%_CO_ at 500, 200 and 100 mA cm^-2^, respectively). For CO mol% > 50, only net CO consumption was apparent over the entire range of current density. This data allowed us to estimate what fraction of the produced C_2+_ species can be accounted for by an exclusive CORR pathway. To do that, we considered the *apparent CO reduction reaction selectivity*, S_CORR_, defined as the ratio of the experimental CO consumption rate, CO_consumption_ = –CO_net flux_ and the theoretical CO demand that is required for the combined experimental C_2+_ production rate, according to:

1 $${S}_{{CORR}}=\frac{{{{{{{\rm{CO}}}}}}}_{{{{{{\rm{consumption}}}}}}}}{\mathop{\sum}\limits_{i}({\dot{n}}_{i}\,{\nu }_{i})}\,\cdot 100\%$$

where ${\nu }_{i}$ and ${\dot{n}}_{i}$ denote the number of carbon atoms in species i and its molar production rate, respectively.

Figure 3c plots *S*_*CORR*_ versus the co-feed compositions and the applied current density. Both CO mol% in the feed and applied current density control S_CORR_, with S_CORR_ approaching 100% with larger applied current density or with rising CO mol%. As expected, a pure CO feed showed a S_CORR_ of 100% at all currents, agreeing with the conditions of CO being the only reactant. Importantly, sustained S_CORR_ values near 100% were evident for 67 mol% CO feed and currents as low as 200 mA cm^−2^. This observation suggests that under conditions of 67 mol% CO and 200 mA cm^-2^ essentially all C_2+_ product formation can be accounted for by the reduction and dimerization of CO gas (prevalent CORR). This kinetic effect arises because the catalytically effective gas composition at the catalyst-electrolyte-interface differs from the bulk gas feed composition. It is a consequence of the preferential acid-base reaction of CO_2_ in CO_2_/CO co-feeds with catalytically generated OH^−^^18,19^. This acid-base reaction increases the effective CO concentration for a given CO_2_/CO co-feed, and this will become more severe at a larger current. Using S_CORR_, we split the catalytic co-feed reduction kinetics into a CO-controlled regime (S_CORR_ > 50%) and a CO_2_-controlled one (S_CORR_ < 50%), see colored regions in Fig. 3c, which deconvolutes the overall reduction reactivity.

### Carbon Selectivity of C_2+_ production under CO_2_/CO co-feeds

Rising CO concentration in CO_2_/CO co-feeds sharply enhances the total production rates of C_2+_ species (Fig. 2). In part, this influence could be explained by the reduced number of electrons required to produce C_2+_ species from the CORR pathway, see Supplemental Discussion 1. However, charge stoichiometry falls short of the 2 to 4-fold experimental increase shown in Fig. 2. This is manifested in the single feed experiments CO_2_RR and CORR in Supplementary Fig. 4 that showed drastically larger FE values for C_2+_ species in CORR experiments: In particular, the FE values of alcoholic oxygenates showed sharp increase under CORR compared to CO_2_RR^14,16^. We hypothesize that the sharp rise in the C_2+_ production must originate from a change in surface catalytic selectivity associated with an enrichment of CO gas in the feed inside the electrolyzer, and associated with a changed surface coverage of CO at the Cu catalyst. Accurate calculation of faradaic product selectivity requires knowledge of the electron transfer numbers, which, however, are not known for co-feeding. This is why, for the purpose of a mechanistic discussion, we calculated a *carbon selectivity* of species i, *CS*_*i*_, (see Supplementary Fig. 6) defined as the ratio between the carbon atom flux into an individual C_2+_ species i and the total carbon atom flux into all C_2+_ products.

2 $${{{{{{\rm{CS}}}}}}}_{{{{{{\rm{i}}}}}}}=\frac{{\dot{n}}_{i}{\nu }_{i}}{\mathop{\sum}\limits_{i}({\dot{n}}_{i}\,{\nu }_{i})}\cdot 100\%$$

Here, ${\dot{n}}_{i}$ and ${\nu }_{i}$ denote the molar production rate of species i and the number of carbon atoms in species i, respectively. *CS*_*i*_ reveals changes in mechanistic reaction pathways for different co-feeds and currents and can be compared to kinetic modeling results. More detailed information on *CS*_*i*_ is given in the Supplemental Discussion 1.

In Supplementary Fig. 6, CS_ethylene_ showed a maximum at 33 CO mol% and decreased at higher CO mol% in the feed. Above 10 mol%_CO_, CS_EtOH_ rose gradually by ca. 10 percentage points, and so did CS_PrOH_. Evidently, higher CO mol% favor formation of oxygenates. While the applied current density showed a minor effect on CS_Ethylene_, the production of either alcohol was profoundly affected: Higher currents favor the production of EtOH over PrOH. Mechanistically, this can be rationalized by a lower surface coverage of CO that makes formation of C_3_ products less likely, as concluded in prior experiments of pure CO feeds at varying partial pressures^12,13,44–46^. A more detailed mechanistic discussion of the observed trends in *CS*_*i*_ values under CO_2_/CO co-feeds is provided in the Supplemental Discussion 2.

### Operation of a two-cell, tandem system for enhanced production rates and energy efficiency of C_2+_ compounds

After we had deconvoluted and rationalized the increase in overall C_2+_ production rates under controlled CO_2_/CO co-feed electrolysis, we moved to assemble, operate, and analyze the first complete low-temperature, coupled CO_2_ tandem electrolyzer cell system excluding intermediate processing steps. Unlike *tandem catalyst* designs^20^, *tandem cell* designs allow for individually optimized active sites and electrolysis conditions for each step. We generated CO-rich co-feeds in “Cell-1” using a neutral-pH, zero-gap electrolyzer with a Ni-N-C catalyst-based Gas Diffusion Electrode (GDE)^43^. The use of the CO_2_-to-CO electrolyzer as cell-1 allowed precise control over the resulting mixed CO_2_/CO exit gas composition (equivalent down-stream to the CO_2_/CO co-feed into cell−2) via two control parameters, that is the applied current density and the CO_2_ feed rate.

As operating conditions of cell-1, we selected 200 mA cm^−2^ and near-100% FE_CO_ including only minute amounts of H_2_ to achieve a controlled mixed of predominantly CO_2_/CO exit steam out of cell-1. Supplementary Fig. 7 shows how the FE_CO_ affects the absolute CO production rate as a function of the applied current density of cell-1. At the chosen operating point, cell-1 generates about 7.6 ml_CO_ min^-1^. To ensure stable performance of cell-1, we monitored the experimental FE_CO_ and cell potential over the electrolysis time of 12 hours at 200 mA cm^−2^, shown in Supplementary Fig. 8. We thereby convinced ourselves that the FE_CO_ value remained at near-unity without changes in the cell potential. Since the CO_2_ flow rate into cell-1 controls the molar CO_2_/CO ratio of the exit steam, we have also investigated the effect of CO_2_ flow rate. We chose (i) a flow rate of 50 ml min^−1^ corresponding to the total flow rate of the co-feed tests above, and (ii) a reduced CO_2_ flow rate of 30 ml min^−1^ to enrich the exit flow in CO further. In cell-1 the CO_2_ flow of 50 mL min^−1^ was converted to a gas mixture (co-feed) of CO_2_ and CO with 18 mol% of CO and a total volumetric flow rate of 42 mL min^−1^, whereas a CO_2_ input of 30 mL min^−1^ resulted in an exit flow of 22 mL min^−1^ with 35 mol% of CO.

**Fig. 4:**
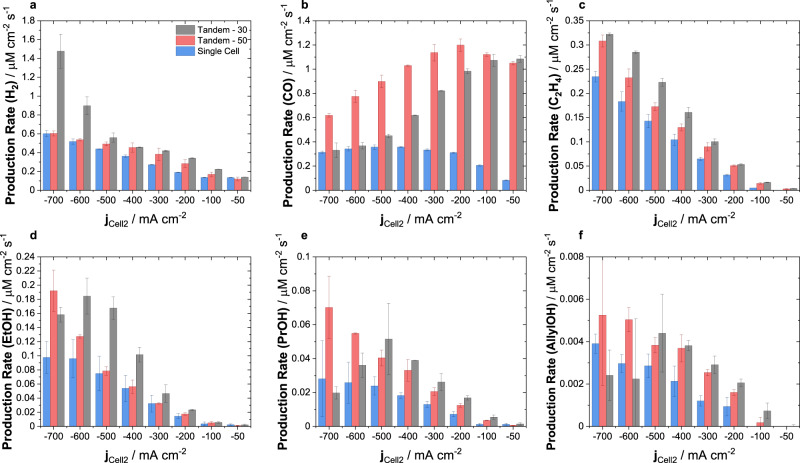
Product production rate comparison of tandem electrolyzer cell cascade (cell-1 and cell-2) versus a single electrolyzer cell. Production rate of **a** hydrogen, **b** carbon monoxide, **c** ethylene, **d** ethanol, **e** n-propanol, and **f** allyl alcohol as a function of current density applied to cell-2. Values given in the legend correspond to the volumetric flow of CO_2_ introduced to the tandem system. In the tandem configuration, cell-1 has been continually operated at a current density of 200 mA cm^−2^ while the current density applied to cell-2 has been varied. For both cells the active geometric surface area is 5 cm^2^. The single-cell refers to CO_2_RR experiments using only cell-2 of the reaction cascade at a CO_2_ volume flow of 50 mL min^−1^. Displayed values represent the average and error bars the standard deviation of at least 2 independent measurements.

Figure 4 compares the catalytic production rates of the tandem system versus the reference single-cell CO_2_RR (using cell-2) as a function of the applied current density of cell-2. The investigated CO_2_ flow rates of 50 mL min^−1^ and 30 mL min^−1^ are denoted as *Tandem-50* and *Tandem-30*, respectively, the reference is denoted as *Single Cell*. In good agreement with our previous experiments, hydrogen production slightly increased with more CO in the co-feed between cell-1 and cell-2, Fig. 4a. Tandem-30 showed a larger H_2_ production rate than Tandem-50, consistent with its larger CO mol% in the exit of cell-1. While Tandem-50 matched the H_2_ production rate of the single-cell experiment at 700 mA cm^−2^, H_2_ rate of Tandem-30 increased rapidly at larger current densities, most likely due to reactant transport limitation. *CO production rates* of Tandem-50/30 at 50 mA cm^−2^, Fig. 4b, are controlled by CO production of cell-1. CO rates of Tandem-30 declined steadily approaching the Single Cell, while CO rates of Tandem-50 showed a maximum at 200 mA cm^−2^ before declining. *Ethylene rates*, Fig. 4c, were significantly enhanced (up to 50% at 500 mA cm^−2^) for the Tandem case over the entire current density range. A similar behavior was seen in the *EtOH production rates*, Fig. 4d, with space-time yield enhancements of up to 100% at 700 mA cm^−2^. Likewise, *production rates of C*_*3*_ *compounds*, i.e., PrOH and AllylOH, Fig. 4e, f, showed more than 100% enhanced production rates at 700 mA cm^−2^ in tandem configuration, with Tandem-30 suffering from mass transport limitations.

The experimental performance of the tandem systems are in good agreement with our CO_2_/CO co-feeds results. Tandems showed a sharp increase in C_2+_ production rates over the single-cell CO_2_RR experiments. While C_2+_ production rates of Tandem-30 were superior to Tandem-50, consistent with the larger CO concentration in the co-feed to cell-2, clear signs of mass transport limitation were apparent when exceeding a current density of 500 mA cm^−2^, reflected in a rising H_2_ rate and a declining C_2+_ rate. Note that the extent of the decrease in C_2+_ rates at large current density followed the order of PrOH/AllylOH>EtOH>C_2_H_4_ suggesting that C_3_ compounds are more susceptible to the insufficient reactant transport. This behavior fits well with our previous observation in single-cell experiments that showed that C_3_ production rates were particularly sensitive to CO depletion in the gas feed.

In order to provide a direct comparison of product productions in the tandem experiments (18 mol%_CO_ for Tandem-50, and 35 mol%_CO_ for Tandem-30) and the CO_2_/CO co-feed experiments, we overlaid their production rates in Supplementary Fig. 9. This revealed some unexpected, yet important kinetic discrepancies: In either tandem experiment, the observed production rates suggested a higher CO mol% in the co-feed to cell-2 than inferred from the overlaid co-feed single-cell experiments. This trend is also reflected in the C_2+_ product carbon selectivity comparisons in Supplementary Fig. 10. With larger current densities, this seeming CO-enrichment becomes progressively more pronounced. To explain this observation, we recall the lower actual tandem feed rates of 42 mL min^−1^ and 22 mL min^−1^ for Tandem-50 and Tandem-30, respectively, compared to 50 mL min^−1^ for the single cell case. This difference in available CO_2_ becomes clearer in a direct comparison of the molar reactant flow to cell-2 under the various conditions, see Supplementary Fig. 11. As a result of this, at a given OH^-^ generation (given current density), the lower molar CO_2_ flow into cell-2 results in a larger share of CO_2_ being depleted. This enhances the effective CO concentration near the electrode, and results in a product pattern resembling a higher bulk mol%_CO_.

The technological application of the tandem concept ultimately requires an acceptable overall *energy efficiency*, henceforth denoted *EE*. Estimation of EE values in a tandem system requires power-in /power out consideration of both cells of the reaction cascade. Equation (3) defines the EE as the ratio between the rate of chemical energy stored in products based on their higher heating values (HHV) and the total electrical input power introduced to the electrolyzer system.

3 $${{{{{{\rm{EE}}}}}}}_{{{{{{\rm{i}}}}}}}\left({{{{{\rm{HHV}}}}}}\right)=\frac{{\dot{n}}_{i}\,{{{{{{\rm{HHV}}}}}}}_{{{{{{\rm{i}}}}}}}}{{I}_{{Cell}1}*{E}_{{Cell}1}+{I}_{{Cell}2}*{E}_{{Cell}2}}*100\%$$

**Fig. 5:**
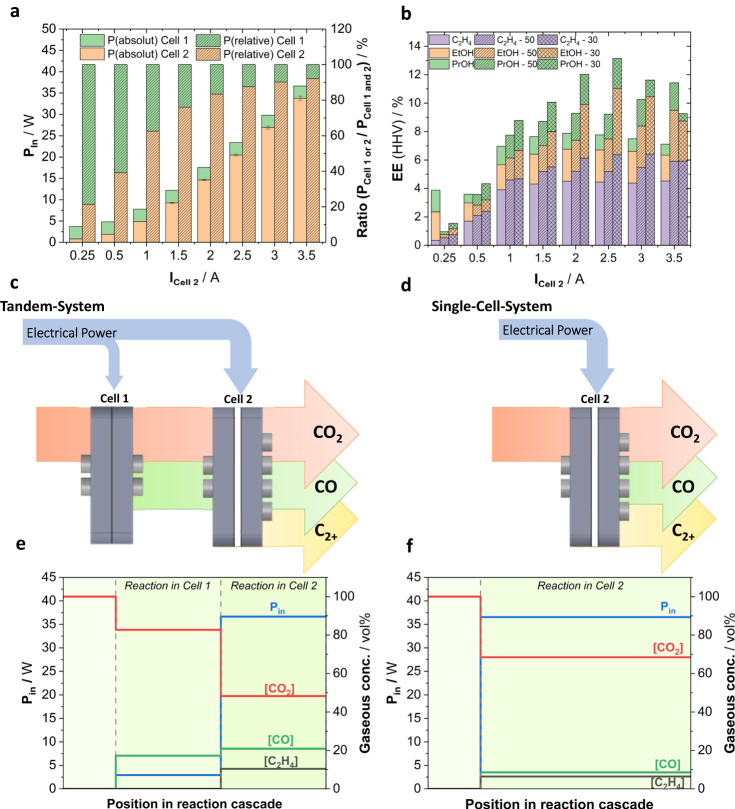
Efficiency and system characteristics comparison between single-cell and tandem experiments. **a** Total input Power, P_in_, as function of applied current to cell-2 deconvoluted in absolute and relative terms into the individual contributions of cell-1 and cell−2 during tandem experiments with CO_2_ input flow of 50 mL min^−1^. **b** Energy efficiency for C_2_H_4_, EtOH and n-PrOH based on their higher heating value (HHV) as a function of applied current for single-cell and tandem experiments. Tandem experiments are labeled in the legend with the respective input flow of CO_2_ (30 or 50 mL min^−1^). **c**, **d** Schematic illustrations of c the tandem electrolyzer cell system and d single-cell system with arrows indicating the flow of products and reactants, CO_2_, CO and C_2+_, as well as the distribution of electrical power across the coupled electrochemical cells. **e**, **f** Distribution profiles of total power input and concentrations of CO_2_, CO and C_2_H_4_ within the reaction cascade for e tandem experiments with 50 mL min^−1^ CO_2_ feed and f single-cell experiments. In the tandem configuration, cell-1 has been continually operated at a current density of 200 mA cm^−2^ while the current density applied to cell-2 has been varied. For both cells the active geometric surface area is 5 cm^2^. Displayed error bars represent the standard deviation of at least 2 independent measurements.

In Fig. 5, we compare the performance of the single-cell and tandem system experiments with particular focus on the electrical power consumption, energy efficiency, and system characteristics. Figure 5a deconvolutes the electrical input power, P_in_, of the Tandem-50 system in terms of cell-1 and cell−2 in absolute and relative terms. As cell-1 was operated at constant current and cell voltage, absolute P_in_ of cell-1 remained constant, while P_in_ of cell-2 strongly increased. This has important implications for the relative P_in_ balance between the two cells: At low currents applied to cell-2, total P_in_ is clearly dominated by cell-1, later by cell−2 increasing to over 90% at 3.5 A. This is because of the higher applied currents and cell resistances of the 1-gap design of cell−2 compared to the zero-gap design of cell-1.

Figure 5b plots the EE of C_2_H_4_, EtOH and PrOH in Tandem-50 and Tandem-30 versus the single-cell design. While at low currents the single-cell system shows competitive C_2+_ EE values, at larger currents > 1 A tandem designs sharply outperform the single-cell system. In particular, the Tandem-30 experiments shows a strongly enhanced total absolute C_2+_ EE of up to 13%. We then analyzed the individual EE of C_2_H_4_, EtOH and PrOH as a function of total P_in_ for each electrolyzer design in Supplementary Fig. 12. The tandem systems showed relative enhancements in C_2+_ EE values of up to 100% over a broad operation range, in particular Tandem-50 at high C_2+_ productivities and large power inputs. At low power input, the productivity of the tandem system was dominated by cell-1 reflected in the high EE for CO and low EE for H_2_ in comparison to the single-cell system shown in Supplementary Fig. 13. In addition to the advantages for EE, we also observed a more efficient reactant utilization described by the single pass carbon efficiency, SPCE. When directly comparing the single-cell system to the tandem-system, the SPCE nearly doubled from 17% to 30–35% depending on the applied current, see Supplementary Fig. 14. The increase in SPCE was larger than can be expected by a simple consideration of the additional operation of cell-1 suggesting the inherent benefit of tandem operation. A more detailed discussion can be found in the Supplementary Information of this work.

Apart from the electrochemical performance differences, tandem electrolyzer systems also differ from a single-cell system in terms of some of their process characteristics. As the single-cell system deploys only one single reactor, the total input power, the CO_2_ consumption, and product generation occur at the same physical location, schematically depicted in Fig. 5d. This results in a single-stepped experimental concentration and power profile, as given for 700 mA cm^−2^ in Fig. 5f. By contrast, in the tandem system the total input power splits into two reactors and the conversion of CO_2_ occurs along a reaction cascade, schematically depicted in Fig. 5c. Consequentially, the experimental concentration and power profile at 700 mA cm^−2^ (cell-2), given in Fig. 5e, reveals two distinct steps.

In summary, we have designed, assembled, and analyzed the first full low-temperature tandem electrolyzer cell system designed for an efficient electrochemical CO_2_ reduction to C_2+_ products, such as Ethylene, EtOH, and PrOH. The tandem design consisted of two low-temperature electrolyzer cells in series, which enabled a catalytic reaction cascade of the reaction of CO_2_ to mixed CO_2_/CO streams in cell-1, coupled to the reaction of mixed CO_2_/CO feeds to C_2+_ products in cell-2. We demonstrated that the tandem system strongly enhanced production rates of C_2+_ compounds compared to the use of a conventional singe-cell system. We also provided evidence that the additional power input required to operate the tandem system was comperatively minor thanks to the drastically lower energetic demand of the CO_2_-to-CO electrolyzer cell-1. Consequentially, the proposed tandem system allowed for an increase in individual EE of C_2+_ species compared to the conventional single-cell system.

## Methods

### Electrochemical cell and electrode preparation

Depending on the target product of electrolysis, electrochemical tests were performed using two different cell geometries. In the case of CO_2_-to-CO reduction, a “zero-gap” cell was used shown in Supplementary Fig. 2a, referred to as cell-1. For the reduction of either CO_2_ or CO, or co-feed mixtures of the two to produce C_2+_ species a “one-gap” cell was used, shown in Supplementary Fig. 2b, referred to as cell-2.

#### Details on cell-1

The Ni-N-C catalyst used in this work was synthesized through thermal carbonization of a Ni-imidazolate according to a previously established procedure. In short, to an aqueous solution of Ni(NO_3_)_2_ and imidazole, a NaOH solution was added dropwise. After overnight stirring, the precipitate was filtered, washed, and freeze-dried. This catalyst precursor material was then carbonized by thermal treatment in N_2_ atmosphere at 800 °C. The crude product was acid-washed at 80 °C in H_2_SO_4_, followed by rinsing with water to pH-neutral and freeze-drying to obtain the final catalyst powder^43^. For the preparation of the cathode GDE, 55 mg as-prepared Ni-N-C catalyst, 195 mg Sustainion solution (Dioxide Materials, 5 wt% Sustainion in ethanol solution), 100 μL DI-water and 2900 μL isopropanol were mixed and sonificated using a sonifer horn for 15 mins. Afterwards, the prepared ink was sprayed coated at 60 °C onto 5 cm^2^ of the micro porous side of a commercial gas diffusion layer provided by DeNora (GDL2). As electrochemical cell, a membrane electrode assembly type electrolyzer setup was deployed for the CO_2_ to CO conversion. An exploded-view of the electrolyzer system is given in Supplementary Fig. 2a. The catalyst-coated cathode GDL, membrane (Sustainion membrane X37-50 RT, Dioxide material), and the anode (5 cm^2^, commercial IrO_2_-GDE supplied from Dioxide Materials) were assembled layer by layer including PTFE gaskets (thickness: 200 microns; window size: 5c m^2^) to guarantee for leak-tightness. Finally, all layers were compressed between the electrolyzer endplates, which served as flow fields and current collectors. For catalytic tests involving cell-1, an aqueous solution of 0.1 M KHCO_3_ electrolyte was used on the anode side only and constantly recycled at a volumetric flow rate of 20 mL min^−1^. On the cathode, a humidified stream of CO_2_ was introduced by a mass flow controller at a rate of 25 mL min^−1^. After the reaction in cell-1, the product stream, composed mainly of CO and unreacted CO_2_, was introduced as reactant gas feed to the cathode GDE of cell-2.

#### Details on cell-2

As anode material Ni foam (Fraunhofer IFAM, thickness of 0.45 mm) was placed inside the flow field of the anode Titanium endplate (Dioxide materials). For the preparation of the cathode GDE a sintered PTFE membrane (Elringklinger, thickness of 0.5 mm) was used as substrate and coated by spray-painting with a dispersion of commercial spherical Cu particles (Sigma-Aldrich, 40–60 nm) and PTFE particles (Sigma-Aldrich, 1 μm) in ethanol. The relative content of PTFE particles was adjusted to achieve a final loading of 20 wt.% in relation to the combined loading of Cu and PTFE particles on the substrate. The absolute loading of Cu particles on the PTFE substrate was controlled by the volume of the catalyst dispersion used during the spray-painting process and fixed at 3.0 mg cm^−2^ throughout the whole study. When assembling the electrolysis cell, the Cu-PTFE GDE is fixed to the cathode Titanium endplate with a conductive Cu tape to enable electronic contacting of the catalyst layer. Silicon gaskets of 0.5 mm in thickness and a cut-out window of 5 cm^2^ were used to expose 5 cm^2^ of the electrode as geometric active area and guarantee a leak-free operation during catalytic testing. An aqueous solution of 1.0 M KHCO_3_ with a volume of 500 mL each was deployed as anolyte and catholyte solution. In the cathode compartment, a custom-designed flow field composed of PEEK and a thickness of 1.0 mm was deployed. The anode and cathode compartments of the electrochemical cell were separated by an anion exchange membrane (Selemion, AMV).

### Electrochemical setup

For electrochemical testing the electrolysis cell was embedded in the teststand depicted in Supplementary Fig. 2c that allowed careful control over pressure levels, as well as gas and electrolyte flow rates. Mass flow controllers (Bronkhorst) were used to accurately adjust the flow of all gases (N_2_, CO, CO_2_). The reactant gas feeds, CO_2_ and CO pure feeds or gas mixtures of the two, were introduced at a total constant rate of flow of 50 mL min^−1^. The anolyte and catholyte were constantly recirculated through the respective compartments of the cell by membrane pumps (KNF, SIMDOS 10) at a constant flow of 50 mL min^−1^. Additionally, a dead volume filled with gas is induced on the inlets and outlets of the membrane pumps to mitigate their inherent pulsation during operation. The cell was operated with an overpressure on the liquid side of the cathode GDE. Here, the differential pressure across the cathode was set to 100 mbar by adjusting the absolute pressure of the cathode gas side to 1.300 bar and the absolute pressure of the catholyte reservoir headspace to 1.400 bar by use of back pressure regulators (Bronkhorst).

### Electrochemical measurement protocol

Electrochemical characterization for cell-2 was carried out according to a preset measurement protocol comprising three successive measurement techniques: Firstly, galvanostatic steps in current density, secondly cyclovoltammetry (CV) and finally galvanostatic electrochemical impedance spectroscopy (GEIS). All electrochemical data that show error bars represent the mean values and standard deviation obtained from at least two independent samples.

#### Galvanostatic current density steps

Here, after an initial equilibration time of 20 min at open circuit potential (OCP), the cathodic current density was increased stepwise as follows: 50, 100, 200, 300, 400, 500, 600 and finally 700 mA cm^−2^. After the 700 mA cm^−2^ current density step, the current was decreased again, retracing the previous steps in the opposite direction towards a final current density of 50 mA cm^−2^. During the whole procedure, each of the set current density steps was kept constant for a period of 20 min before moving towards the next value.

#### Cyclovoltammetry (CV)

After completion of the galvanostatic current density steps, CVs were conducted in a potential window of 100 mV (−0.600 to −0.700 V_Ag/AgCl_). Within this potential window 5 CVs were conducted at a constant scan rate before increasing the scan rate and performing 5 CVs again. This procedure was performed sequentially for scan rates of 1, 2, 5, 10, 20, 50, 100, 200, 500, 700, 1000, 1500 and 2000 mV s^−1^.

#### Galvanostatic electrochemical impedance spectroscopy (GEIS)

In the final step, GEIS measurements were conducted at 1, 10, 20, 50, 100, 200, 300, 400, 500, 600, and 700 mA cm^−2^ of absolute cathodic current. The frequency was varied between 500 kHz and 100 mHz and the amplitude of the interference signal was set to 10% of the respective current density value at which the GEIS measurement was carried out.

### Quantification of products

All data that show error bars represent the mean values and standard deviation obtained from at least two independent samples.

#### Gaseous products

After electrochemical reaction in the electrolyzer the outgoing gas stream was mixed with a constant flow of N_2_ as an internal standard (16 mL min^−1^) and CO_2_ (150 mL min^−1^) that has been purged through the headspace of the catholyte compartment in order to collect gaseous products that were released at the catholyte side during operation. This mixture of gases was introduced into a gas chromatograph (Shimadzu, GC 2014 series) in the following denoted as GC. The GC was equipped with a thermal conductivity detector (TCD) used in the quantification of H_2_ and N_2_ concentrations, as well as a flame ionization detector (FID) that was used for quantification of methane, ethylene and CO. For the detection of CO by the FID a methanizer has been used for the thermal reduction of CO to CH_4_ prior to its detection. Determination of the absolute volumetric flow after electrolysis has been conducted based on changes in the concentration of N_2_ standard as detected by the TCD.

#### Liquid products

During electrolysis, aliquots of the catholyte have been collected after 20 min of reaction time at constant current. These aliquots were analyzed for their content of alcohols by liquid injection gas chromatography (Shimadzu, GC 2010 series) equipped with an FID. Furthermore, a high-performance liquid chromatograph (Agilent, 1200 series) equipped with a refractive index detector has been deployed for quantification of carbonic acids and their respective salts.

### Supplementary information

Supplementary Information

Peer Review File

## Data Availability

All data supporting the results of this study, are included in the published article or the associated Supplementary Information. Additional information will be made available upon reasonable request to the corresponding author.
