# Supplementary material for: A CO2 electrolyzer tandem cell system for CO2-CO co-feed valorization in a Ni-N-C/Cu-catalyzed reaction cascade
Source: Nat Commun. 2023 Sep 14;14:5680. doi: 10.1038/s41467-023-41278-7 (PMC10502113; doi:10.1038/s41467-023-41278-7)
Supplement: Supplementary file 1 — Supplementary Information [file 41467_2023_41278_MOESM1_ESM.pdf]

Supplementary Information

A CO_2 Electrolyzer Tandem Cell System for CO_2-CO co-feed valorization in a Ni-N-C/Cu-catalyzed Reaction Cascade

Tim Möller [1,‡] Michael Filippi [1,‡] Sven Brückner [1], Wen Ju [1] & Peter Strasser [1]*

[1]The Electrochemical Energy, Catalysis, and Materials Science Laboratory, Department of Chemistry, Chemical Engineering Division, Technical University Berlin, Berlin, Germany

[‡]*These authors contributed equally*

[*]Corresponding author:

Peter Strasser, pstrasser@tu-berlin.de

Supplementary Discussion 1

Difference in electron transfer number for CO_2RR and CORR.

Depending on the reactant molecule, i.e. CO_2 or CO, a varying number of electrons is required for the production of C_{2+} species by electrolysis. The half-cell reaction equations below specify the number of electrons during CO_2RR and CORR required for production of ethylene, ethanol and n-propanol.

CO_2RR towards selected C_{2+} species:

$$2CO_2 + 8H_2O + 12e^- \rightarrow C_2H_4 + 12OH^- \quad (1)$$

$$2CO_2 + 9H_2O + 12e^- \rightarrow C_2H_5OH + 12OH^- \quad (2)$$

$$3CO_2 + 13H_2O + 18e^- \rightarrow C_3H_7OH + 18OH^- \quad (3)$$

CORR towards selected C_{2+} species:

$$2CO + 6H_2O + 8e^- \rightarrow C_2H_4 + 8OH^- \quad (4)$$

$$2CO + 7H_2O + 8e^- \rightarrow C_2H_5OH + 8OH^- \quad (5)$$

$$3CO + 10H_2O + 12e^- \rightarrow C_3H_7OH + 12OH^- \quad (6)$$

Based on the smaller number of electrons, CORR is expected to show larger C_{2+} rates than CO_2RR at constant total currents and comparable competing reactions, such as hydrogen production. Neglecting minor reduction products of CO_2, such as formate or glyoxalic acid, the C_{2+} product production rates are actually proportional to the change in number of required electrons. For the products considered in this study, this decreased demand of electrons would cause an increased production rate of 50 % for all C_{2+} products, i.e. a factor of $\frac{12}{8}$ for C_2H_4/EtOH and a factor of $\frac{18}{12}$ for PrOH.

Definition and Calculation of the carbon selectivity for major C_{2+} species.

As calculation of faradaic selectivity values is not possible in our experiments due to the lack in knowledge over precise values of electron transfer numbers, we introduce and discuss the *carbon selectivity* as a new kinetic descriptor to compare the competing production of C_{2+} species. The carbon selectivity of species i, denoted CS_i, was calculated according to Supplementary Equation 7 that describes the ratio of the molar production of any given C_{2+} species to the total molar production of all C_{2+} species, each weighted by the number of its carbon atoms. Here, $\dot{n}_i$ and ν_i denote the molar production rate of species i and the number of carbon atoms in species i, respectively. Note that we limit our discussion on the major C_{2+} compounds observed in our experiments, i.e. ethylene, ethanol and n-propanol.

$$\mathrm{CS(C_{2+})}_{\mathrm{i}} = \frac{\dot{n}_i \nu_i}{\sum_i (\dot{n}_i \nu_i)} \cdot 100\ \% \quad (7)$$

Supplementary Discussion 2: Mechanistic Discussion of trends in carbon selectivity, CS_i

Key to understanding the catalysis of co-feed experiments is a discussion how the surface coverage of CO varies with increasing CO mol% in the feed stream of a Gas Diffusion Electrode. Prior CO_2RR reports have shown that the adsorption and reduction of CO_2 to CO occurs preferentially at undercoordinated sites, such as steps and kinks. Similarly, selective Pb sub-monolayer deposition on undercoordinated Cu sites suppressed the CO_2RR activity of Cu. [1, 2, 3, 4, 5, 6, 7, 8] This is why Cu steps and kinks are believed to display a higher catalytic activity compared to smooth facets and likely dominate the catalytic CO_2 activation and reduction to adsorbed CO, *CO, and further to adsorbed methyl, $*CH_3$, on Cu. Undercoordinated Cu sites of Cu single crystals or oxide-derived Cu have also been correlated with the dimerization of *CO and subsequent competitive reduction to ethylene.[1, 2, 3, 4, 5, 6, 7, 8, 9] More recently, an alternative ethanol formation pathway was proposed for metallic polycrystalline Cu catalysts based on the dimerization of a methyl intermediate, $*CH_3$, and *CO.[4] Following these mechanistic pathways, it appears conclusive that rising CO mol% in the co-feed result in higher *CO surface coverage, θ_{CO}, which boosts CO dimerization and production of ethylene and EtOH.[10] On the other hand, PrOH formation requires coupling of a C2 and C1 fragment. While PrOH formation may equally benefit from higher θ_{CO} through the coupling of *CO and a *C2O2 dimer, this process is competitive to ethylene and EtOH formation. This why with increasing CO content in the feed up to 50 $mol\%_{CO}$, the $CS_{Ethylene}$ reached a maximum and began to decrease thereafter. In line with the decrease of $CS_{Ethylene}$, both CS_{PrOH} and CS_{EtOH} show a steady increase, consistent with reports on a kinetic favoring of oxygenate production under conditions of high CO surface coverage. [11]

Interestingly, kinetic regimes of CO_2/CO co-feed reduction (Fig. 3b) monotonically increased their CO consumption with rising CO mol% in the feed. Given the vastly different solubility of CO_2 and CO, we consider this observation inconsistent with the simple mechanistic concept of a competition of CO_2 and CO for a shared single catalytic active site on the Cu surface. The experimental data suggest the presence of at least 2 distinct surface sites, one favoring CO_2 activation and reduction to CO, and another favoring CO adsorption and reduction. This concept of reactant-specific Cu surface sites has been recently put forward based on isotope labelling experiments. [11, 12]

Supplementary Discussion 3: Discussion of trends in single pass carbon efficiency, $SPCE_i$

Detrimental in advancing CO_2 electrolysis towards an economical technology, is converting the CO_2 feedstock towards targeted products as efficiently as possible. However, commonly, vastly excessive amounts of CO_2 are used by default to stay well away from regions of reactant depletion and focus on other performance metrics such as the FE for specific products. From a technical view, this is prohibitively decreasing the economic viability of this process, as a large fraction of CO_2 is not converted thereby diluting the product stream and increasing costs of downstream product separation. To quantify and evaluate how efficient the CO_2 (or CO) feedstock has been utilized, the single pass carbon efficiency, referred to as SPCE, has been calculated in agreement with previous reports:

$$\mathrm{SPCE_i} = \frac{\dot{n}_i v_i}{\dot{n}_{feed}(\mathrm{CO_2} + \mathrm{CO})} \tag{8}$$

Where $\mathrm{v_i}$ and $\dot{\mathrm{n}}_\mathrm{i}$ denote the number of carbon atoms in species i and its molar production rate, respectively, and $\dot{\mathrm{n}}_{\mathrm{feed}}(\mathrm{CO_2} + \mathrm{CO})$ refers to the combined molar feed rate of CO_2 and CO reactant deployed during electrolysis.

Supplementary Fig.14 shows the cumulative SPCE of all CO and CO_2 reduction products calculated, respectively, for the various conditions investigated in single-cell and tandem-cell experiments. Generally, the cumulative SPCE observed in single-cell experiments increases with increasing mol% of CO in the feed and, as expected, with increased current density. Only at a lower current density of smaller than 300 mA cm^{-2} a larger CO concentration of the feed did not increase the SPCE, which is correlated to a larger HER observed under these conditions. The highest cumulative SPCE calculated for a pure CO_2 feed in a single-cell experiment was around 17 %, for 700 mA cm^{-2}. While 10 mol% of CO showed a comparable cumulative SPCE, a further increase in CO concentration increased the cumulative SPCE at 700 mA cm^{-2} to 21 % (33 mol%), 24 % (50 mol%), 26 % (67 mol%), and 28 % for a pure CO feed, respectively. For the tandem-cell experiments we obtained a SPCE of 15 % to 29 % and 28 % to 36 % for the CO_2 feeds of 50 sccm and 30 sccm, respectively, depending on the applied current density to cell-2 of the reaction cascade. Note, the SPCE observed for the tandem-system starts out at a higher value but does not increase as strongly with an increase of applied current density (cell-2) comparted to the single-cell system. This behaviour originates in the inherent nature of how the tandem system operates. The initial conversion of CO_2-to-CO of cell-1 dictates the SPCE of the tandem-system at low current of cell-1 and leads to CO being the major reduction product in this current regime. Upon increased current applied to cell-2, other reduction products become dominant that can either originate from CO_2 or CO, however, note that only reduction of CO_2 would further increase the SPCE of the tandem-system. As we demonstrated earlier, the share of products associated to CORR in co-feeds of cell-2 described by S_{CORR} as calculated by us, increases at larger currents. As a result, the major observations for the tandem-system, for one the increase in SPCE with current being more pronounced in a low current regime (cell-2) while approaching a SPCE plateau at larger currents, and this SCPE plateau being approached earlier for the tandem-30 sccm system are in line with our considerations described by S_{CORR} values.

Supplementary Figures

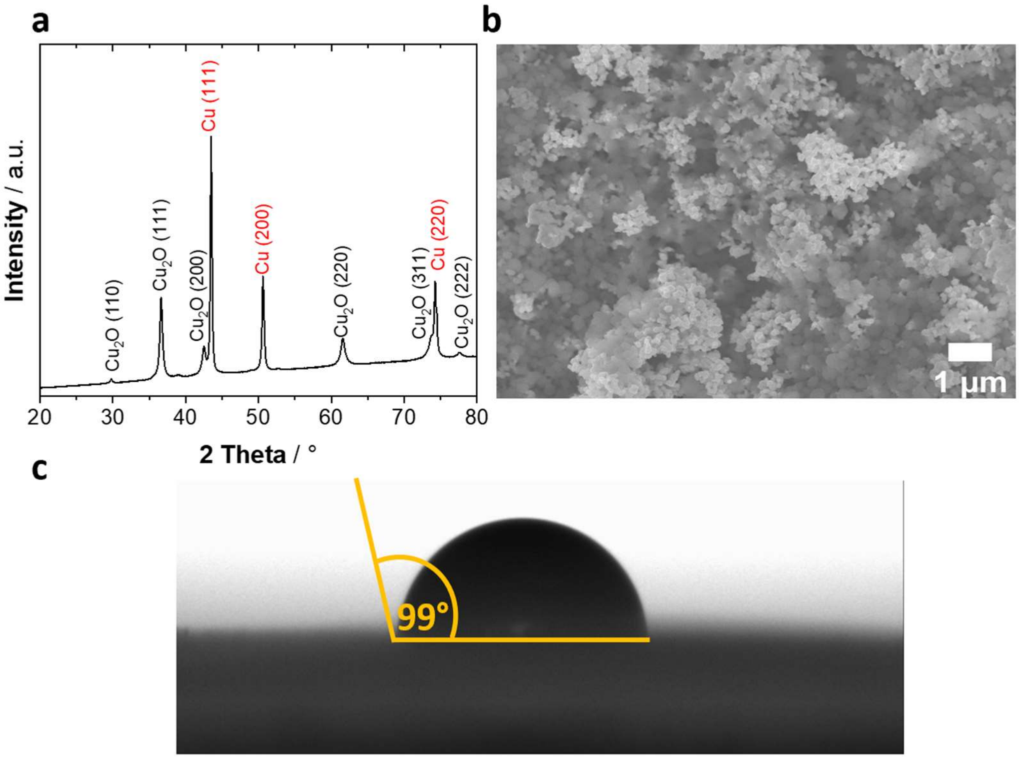

Supplementary Fig. 1 | Cu catalyst and GDE characterization used in cell-2. Material characterisation of the commercial Cu catalysts showing **a** XRD analysis of the catalyst powder, **b** a top-view SEM image of the as-prepared Cu-GDE and **c** a contact angle measurement of the as-prepared Cu-GDE.

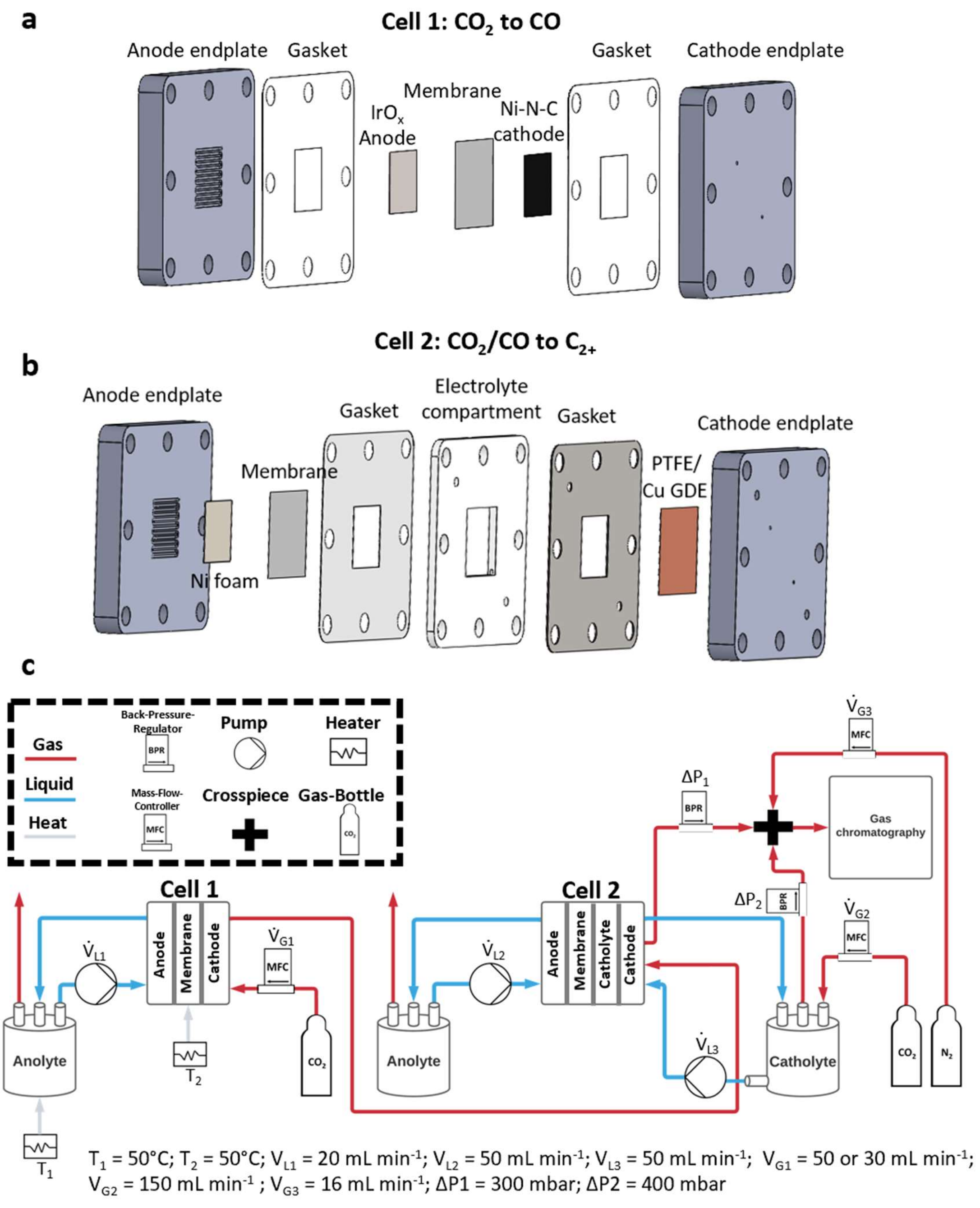

Supplementary Fig. 2 | Illustration of the Electrochemical cell and setup used for catalytic tests. a Exploded-view of the CO_2 to CO electrolyzer, labeled as Cell-1 in tandem experiments. **b** Exploded-view of the CO_2 and/or CO to C_{2+} electrolyzer used in tandem and single cell experiments, generally referred to as Cell-2. **c** Process flow diagram of the setup with embedded Cell-1 and Cell-2 used in tandem experiments.

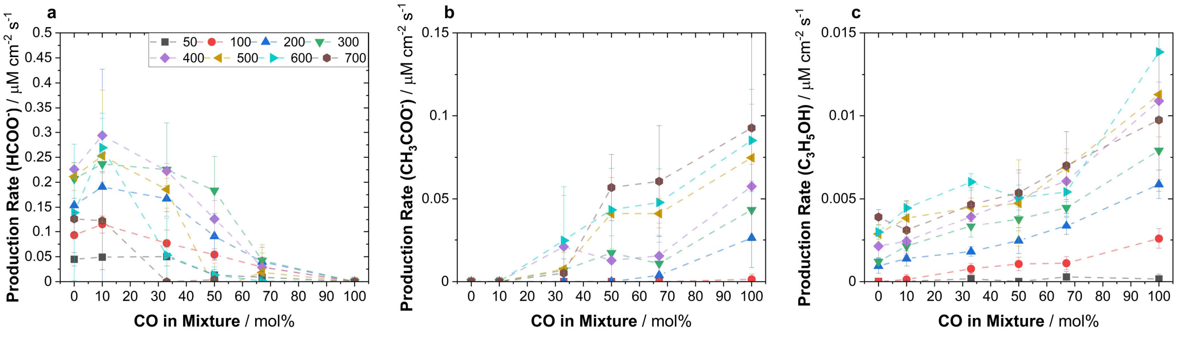

Supplementary Fig. 3 | Production rates of formate ($HCOO^-$), acetate (CH_3COO^-) and allyl alcohol (C_3H_5OH). Production rates of liquid products from electrolysis as a function of CO mol per cent (mol%) in the CO_2/CO co-feed measured for various applied current densities. Production rates are shown for **a** formate, **b** acetate and **c** allyl alcohol. Legend is showing the absolute values of cathodic currents applied. Experiments were conducted at a volumetric flow rate of 50 mL min^{-1} in 1.0 M $KHCO_3$ and a geometric surface are of 5 cm^2. Displayed values represent the average and error bars the standard deviation of at least 2 independent measurements.

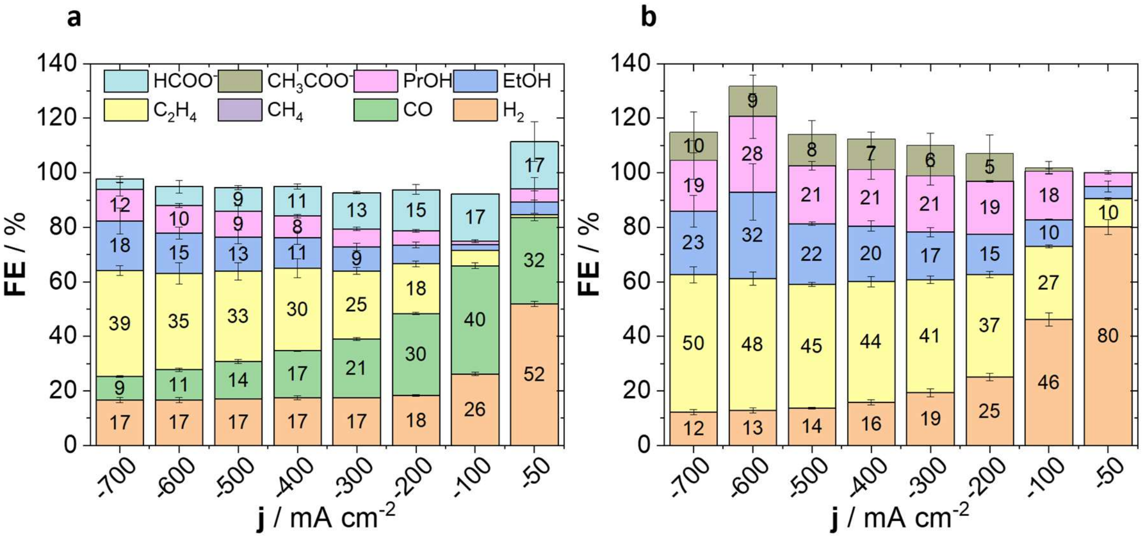

Supplementary Fig. 4 | Selectivity observed for CO_2RR and CORR electrolysis. Faradic efficiency as function of applied current density for single feed experiments conducted in cell-2. Results are shown for **a** CO_2RR and **b** CORR conducted at a volumetric gas flow rate of 50 mL min^{-1} in 1.0 M $KHCO_3$ and exposing a geometric surface area of 5 cm^2. Displayed values represent the average and error bars the standard deviation of at least 2 independent measurements.

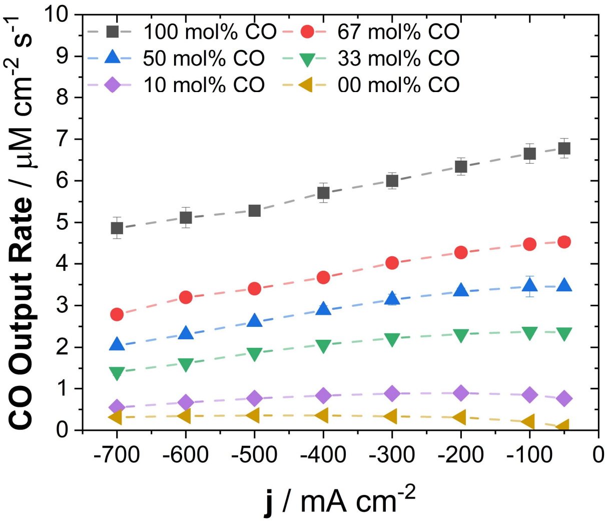

Supplementary Fig. 5 | CO output after co-feed experiments in cell-2. Molar stream of CO gas as detected after electrolysis in cell-2 as a function of applied current density and for various CO_2/CO co-feed compositions. Experiments were conducted at a volumetric flow rate of 50 mL min^{-1} in 1.0 M $KHCO_3$ and exposing a geometric surface area of 5 cm^2. Displayed values represent the average and error bars the standard deviation of at least 2 independent measurements.

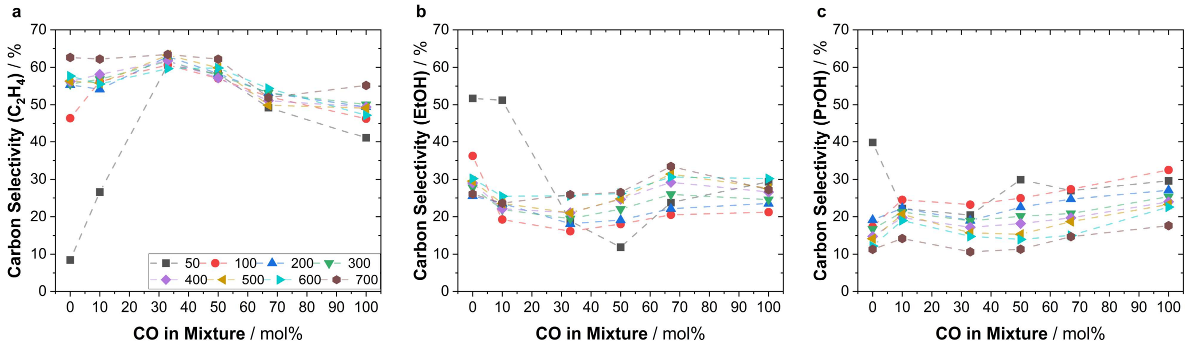

Supplementary Fig. 6 | Carbon Selectivity for major C_{2+} products. Carbon selectivity of **a** ethylene, **b** ethanol and **c** n-propanol as a function of CO concentration in the CO_2/CO co-feed for various currents applied to cell-2. The total volumetric flow rate was kept constant at 50 mL min^{-1} throughout all experiments, while the concentration of CO in a CO_2 gas feed was varied. The active geometric surface area is 5 cm^2.

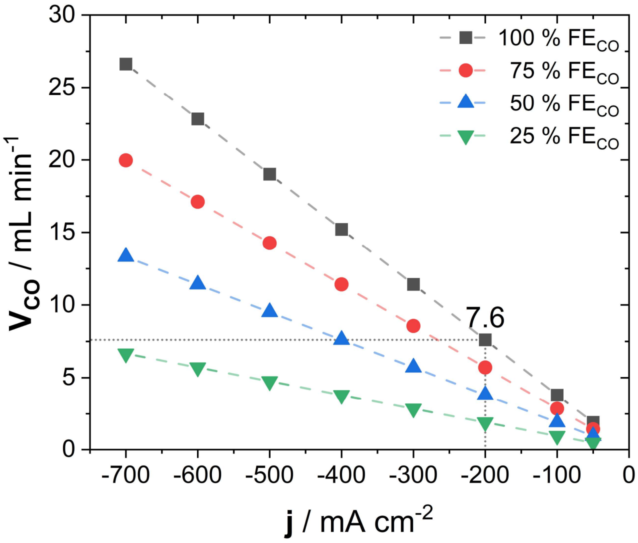

Supplementary Fig. 7 | Theoretical amount of CO produced by cell-1. Theoretical volumetric flow of CO produced as a function of applied current density calculated based on Faraday's law for various FE values for an CO_2-to-CO electrolyzer with a geometric surface area of 5 cm^{-2}. The highlighted value shows the volumetric flow in mL min^{-1} calculated for the case of an applied current of 200 mA cm^{-2} and a CO FE of 100 % in accordance with the operating conditions of cell-1 deployed in this work.

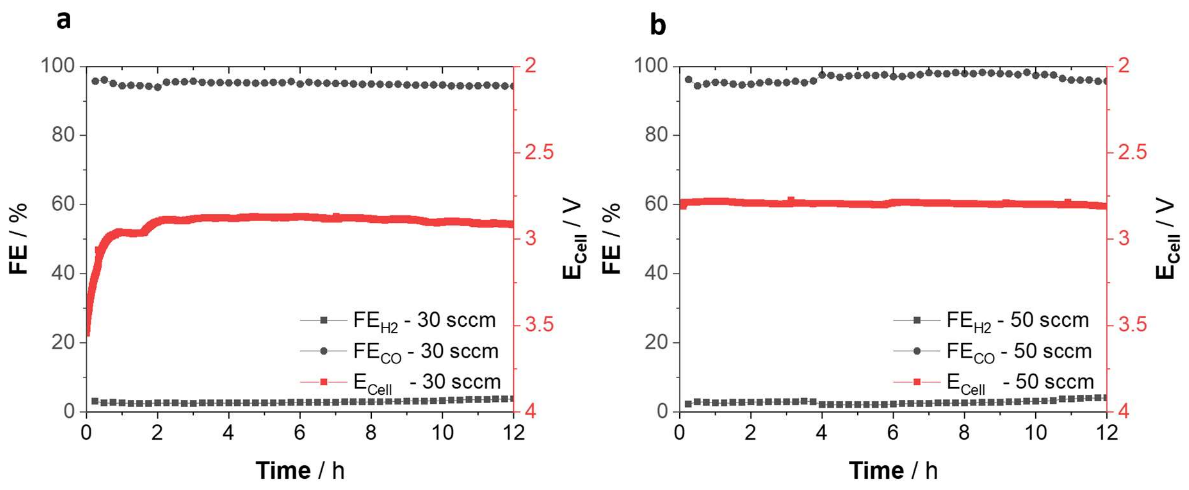

Supplementary Fig. 8 | Electrochemical stability of cell-1. Stability data of CO_2-to-CO electrolyzer, denoted as cell-1, as obtained for an introduced volumetric flow of **a** 30 mL min^{-1} and **b** 50 mL min^{-1} of CO_2 reactant. An aqueous solution of 0.1M $KHCO_3$ was used as anolyte while the cathode was operated with humidified CO_2. The geometric area of the cell was 5 cm^2 and the cell was operated at 50 °C.

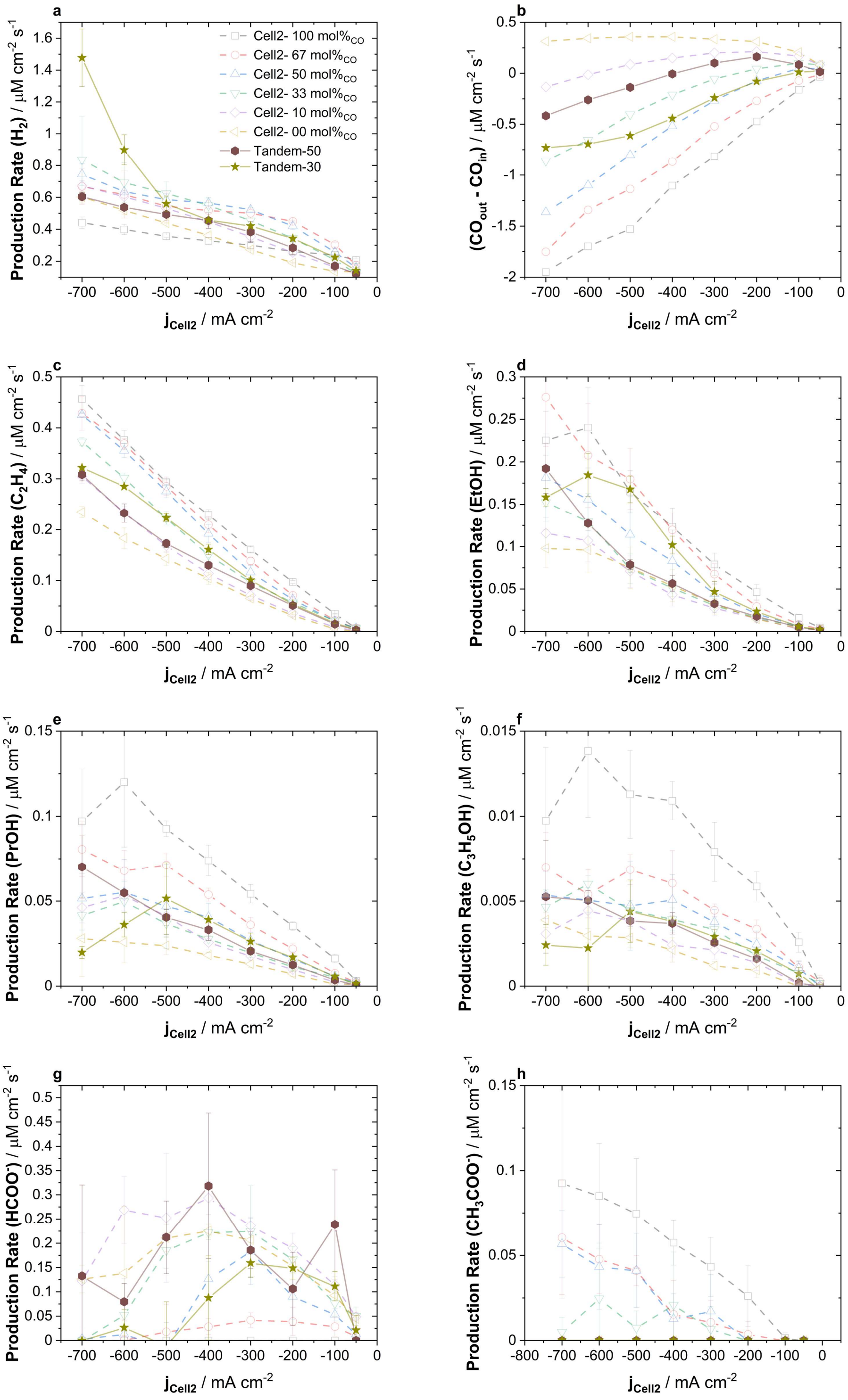

Supplementary Fig. 9 | Comparison of rates observed for various compounds in tandem and single cell systems. Rate of **a** hydrogen, **b** difference in introduced and emitted carbon monoxide, **c** ethylene, **d** ethanol, **e** n-propanol, **f** allyl alcohol, **g** formate, and **h** acetate, as a function of current density applied to cell-2. Tandem values given in the legend correspond to the volumetric flow of CO_2 introduced to the system. In the tandem configuration, cell-1 has been continually operated at a current density of 200 mA cm^{-2} while the current density applied to cell-2 has been varied. In case of single cell experiments, i.e. only use of cell-2, the total volumetric flow rate was kept constant at 50 mL min^{-1} while the concentration of CO in a CO_2 gas feed was varied according to the values given in the legend. For both cells the active geometric surface area is 5 cm^2. Displayed values represent the average and error bars the standard deviation of at least 2 independent measurements.

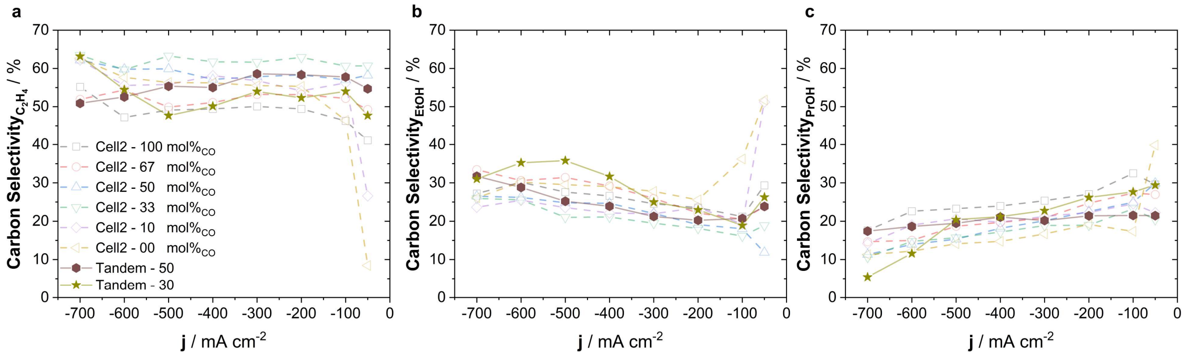

Supplementary Fig. 10 | Comparison of carbon selectivity across major C_{2+} products observed in tandem and single cell systems. Carbon selectivity comparison between **a** ethylene, **b** ethanol and **c** n-propanol as a function of current density applied to cell-2. Tandem values given in the legend correspond to the volumetric flow of CO_2 introduced to the system. In the tandem configuration, cell-1 has been continually operated at a current density of 200 mA cm^{-2} while the current density applied to cell-2 has been varied. In case of single cell experiments, i.e. only use of cell-2, the total volumetric flow rate was kept constant at 50 mL min^{-1} while the concentration of CO in a CO_2 gas feed was varied according to the values given in the legend. For both cells the active geometric surface area is 5 cm^2.

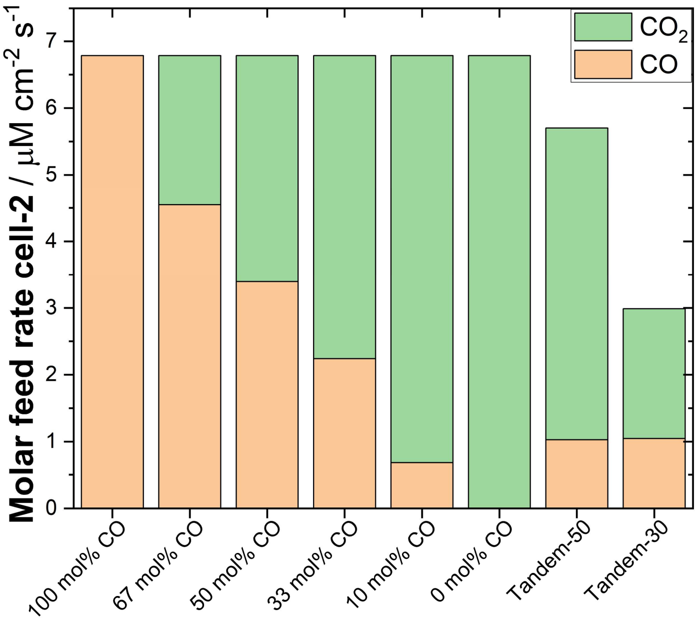

Supplementary Fig. 11 | Reactant feed entering cell-2 under various conditions. Comparison of the molar reactant feed of CO and CO_2 directed to cell-2 in single-cell co-feed experiments and tandem-cell single-feed experiments.

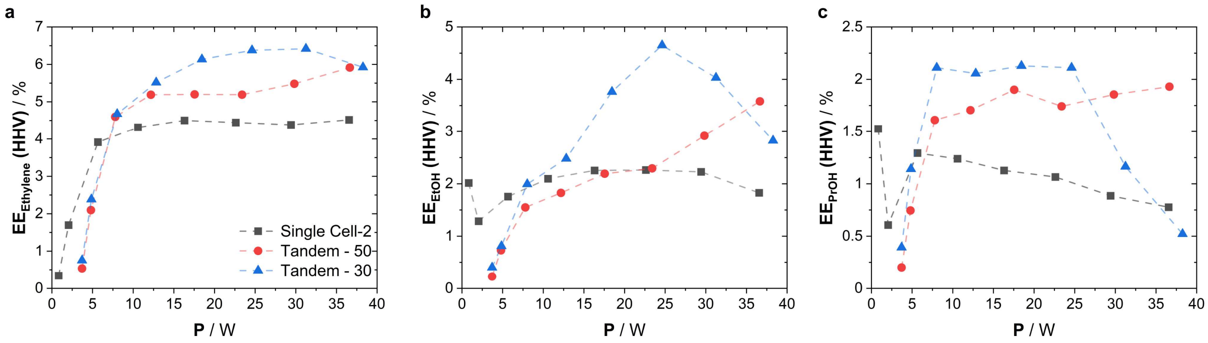

Supplementary Fig. 12 | Comparison of energy efficiencies for selected C_{2+} products. Energy efficiency based on the higher heating values for **a** ethylene, **b** ethanol and **c** propanol as function of total electrical power input for different investigated systems. Values given in the legend correspond to the volumetric flow of CO_2 introduced to the tandem system. In the tandem configuration, cell-1 has been continually operated at a current density of 200 mA cm^{-2} while the current density applied to cell-2 has been varied in between 50 to 700 mA cm^{-2}. For both cells the active geometric surface area is 5 cm^2.

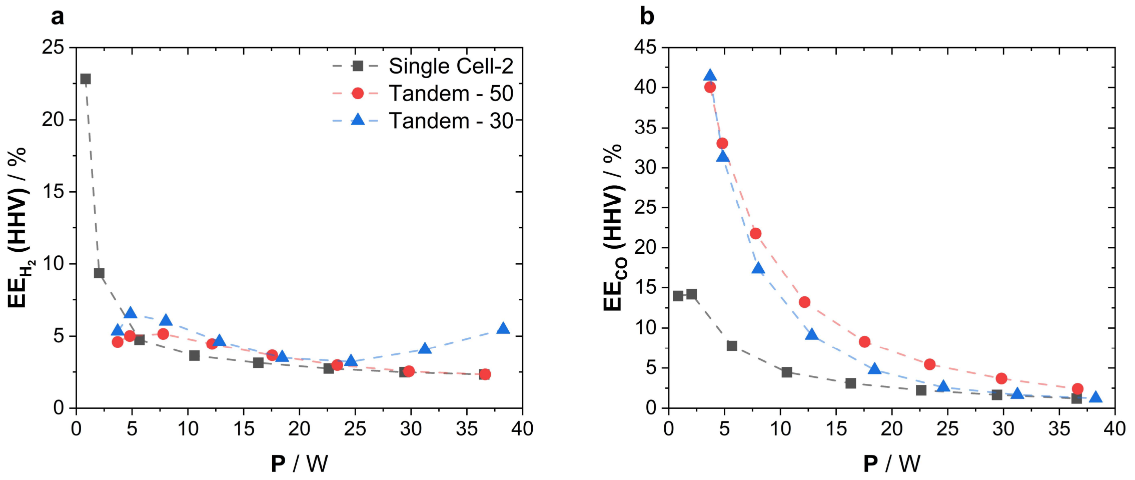

Supplementary Fig. 13 | Comparison of energy efficiencies for hydrogen and CO in tandem and single cell systems. Energy efficiency based on the higher heating values for **a** H_2 and **b** CO as function of total electrical power input for different investigated systems. Values given in the legend correspond to the volumetric flow of CO_2 introduced to the tandem system. In the tandem configuration, cell-1 has been continually operated at a current density of 200 mA cm^{-2} while the current density applied to cell-2 has been varied in between 50 to 700 mA cm^{-2}. For both cells the active geometric surface area is 5 cm^2.

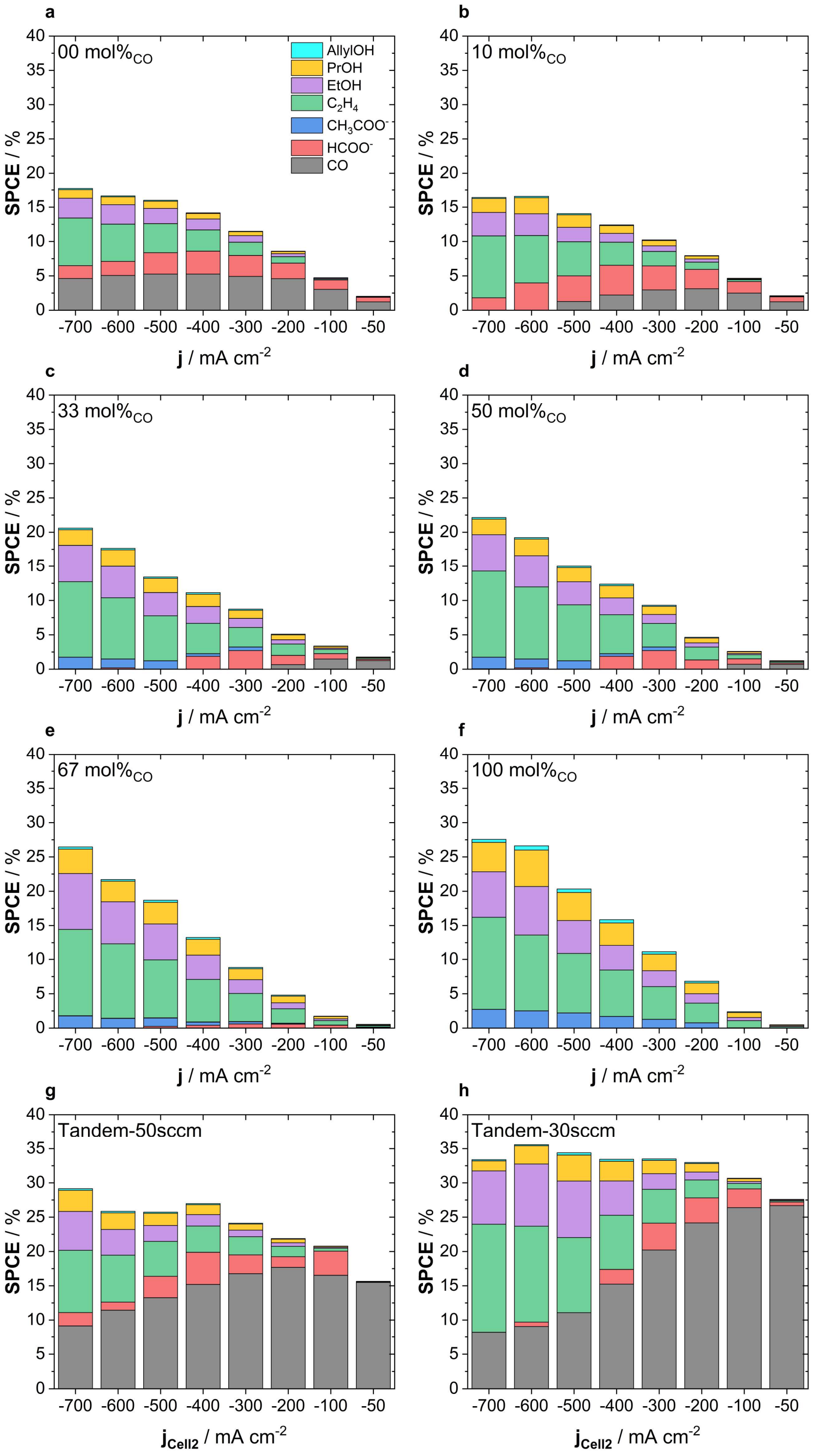

Supplementary Fig. 14 | Comparison of the Single pass carbon efficiency for various investigated systems. Single pass carbon efficiency, referred to as SPCE, divided by product calculated for single-cell experiments at **a** 0 mol%, **b** 10 mol%, **c** 33 mol%, **d** 50 mol%, **e** 67 mol% and **f** 100 mol% of CO in a 50 sccm CO/CO_2 co-feed flow. SPCE is also shown for tandem experiments at **g** 50 sccm and **h** 30 sccm of pure CO_2 feed flow. In the tandem configuration, cell-1 has been continually operated at a current density of 200 mA cm^{-2} while the current density applied to cell-2 has been varied. In case of single cell experiments only cell-2 has been used. Both cell-1 and cell-2 deploy electrodes with a geometric surface area of 5 cm^2.

Supplementary Tables

Catalyst	Cell	Catholyte	GDE Type	Electrode Area [cm^2]	j [mA cm^{-2}]	FE (C2+) [%]	EE (C2+) [%]	SPCE [%]	Ref.
**Cu**	Flow-Cell	7.0 M KOH	PTFE	1	1100	85	20	4.5	[13]
**Cu**	Flow-Cell	3.5 M KOH + 5.0 M KI	PTFE	1	750	~80	N/A	2.2	[14]
**Cu-Al**	Flow-Cell	1.0 M KOH	PTFE	1	400	~90	~ 33 (C_2H_4 half-cell)	N/A	[15]
**CuO**	MEA	0.1 M KOH	Carbon	4	600	~80	N/A	N/A	[16]
**Cu**	MEA	0.1 M $KHCO_3$	PTFE	5	300	~70	~ 21 (C_2H_4)	N/A	[17]
**Cu_2O**	Flow-Cell	1.0 M $KHCO_3$	Carbon	2	300	~55	N/A	N/A	[18]
**Cu**	Flow-Cell	1.0 M $KHCO_3$	Carbon	2	300	~75	N/A	4.2	[19]
**Cu**	Flow-Cell	1.0 M $KHCO_3$	PTFE	5	700	~70	7	18	this work
**Cu**	Flow-Cell	1.0 M H_3PO_4 + 3 M KCl	PTFE	1	1200	~45	N/A	~77	[20]

Supplementary Table 1 | Comparison of various reported conventional CO_2RR electrolyzer systems.

System	Catalyst(s)	Cell and Electrode specifics	Electrode Area [cm^2]	j [mA cm^{-2}]	FE (C2+) [%]	EE (C2+) [%]	SPCE [%]	CO_2 Trap	Ref.
**Tandem-Electrode**	Cu/Ni-N-C	Flow-Cell 1.0 M KOH	1	700	70	N/A	N/A	No	21
**Tandem-Electrode**	Cu/Fe-N-C	Flow-Cell 0.5 M KOH	1	1200	90	17 (C_2H_4)	N/A	No	22
**Tandem-Catalyst**	Cu/Ag	Flow-Cell 1.0 M KOH	N/A	200	50	N/A	N/A	No	23
**Tandem-Catalyst**	CuAg	Flow-Cell 1.0 M KOH	2.5	300	85	N/A	22.5	No	24
**Reaction-Cascade**	Cell-1: Ag	Flow-Cell 1.0 M $KHCO_3$	10	200	22	N/A	N/A	No	25
	Cell-2: Cu	Flow-Cell 1.0 M $KHCO_3$ Catholyte, 2.5 M KOH Anolyte	10	270					
**Reaction-Cascade**	Cell-1: Ag	Flow-Cell 1.0 M $KHCO_3$	10	100	62	N/A	N/A	Yes	25
	Cell-2: Cu	Flow-Cell 1.0 M $KHCO_3$ Catholyte, 2.5 M KOH Anolyte	10	200					
**Reaction-Cascade**	Cell-1: NiO	SOEC 800°C	1.2	550	76	20 (C_2H_4) Only Electricity	11 (C_2H_4)	Yes	26
	Cell-2: Cu	MEA 3.0 M KOH	5	120					
**Reaction-Cascade**	Cell-1: Ni-N-C	Flow-Cell 0.5 M $KHCO_3$	1	140	70	N/A	N/A	Yes	27
	Cell-2: Cu_2O	Flow-Cell 1.0 M $KHCO_3$	1	140					
**Reaction-Cascade**	Cell-1: Ni-N-C	Flow-Cell 0.5 M $KHCO_3$	1	140	60	N/A	N/A	yes	27
	Cell-2: Cu_2O	Flow-Cell 1.0 M $KHCO_3$	2	42.5					
**Reaction-Cascade**	Cell-1: Ni-N-C	MEA 0.1 M $KHCO_3$	5	200	79	11	29	No	This work
	Cell-2: Cu	Flow-Cell 1.0 M $KHCO_3$	5	700					
**Reaction-Cascade**	Cell-1: Ni-N-C	MEA 0.1 M KHCO	5	200	78	13	35	No	This work
	Cell-2: Cu	Flow-Cell 1.0 M $KHCO_3$	5	500					

Supplementary Table 2 | Comparison of various reported CO_2RR electrolyzers that incorporate tandem-systems.

Supplementary References

1. Hahn, C., *et al.* Engineering Cu surfaces for the electrocatalytic conversion of CO2: Controlling selectivity toward oxygenates and hydrocarbons. *Proc. Natl. Acad. Sci. U.S.A.* **114**, 5918-5923 (2017).

2. Arán-Ais, R. M., Scholten, F., Kunze, S., Rizo, R. & Roldan Cuenya, B. The role of in situ generated morphological motifs and Cu(i) species in C2+ product selectivity during CO2 pulsed electroreduction. *Nat. Energy* **5**, 317-325 (2020).

3. Hori, Y., Takahashi, I., Koga, O. & Hoshi, N. Electrochemical reduction of carbon dioxide at various series of copper single crystal electrodes. *J. Mol. Catal. A: Chem.* **199**, 39-47 (2003).

4. Wang, X., *et al.* Morphology and mechanism of highly selective Cu(II) oxide nanosheet catalysts for carbon dioxide electroreduction. *Nat. Commun.* **12**, 794 (2021).

5. Bagger, A., Ju, W., Varela, A. S., Strasser, P. & Rossmeisl, J. Electrochemical CO2 Reduction: Classifying Cu Facets. *ACS Catal.* **9**, 7894-7899 (2019).

6. Wang, L., *et al.* Electrochemical Carbon Monoxide Reduction on Polycrystalline Copper: Effects of Potential, Pressure, and pH on Selectivity toward Multicarbon and Oxygenated Products. *ACS Catal.* **8**, 7445-7454 (2018).

7. Kim, C., Möller, T., Schmidt, J., Thomas, A. & Strasser, P. Suppression of Competing Reaction Channels by Pb Adatom Decoration of Catalytically Active Cu Surfaces During CO2 Electroreduction. *ACS Catal.* **9**, 1482-1488 (2018).

8. Gu, Z., *et al.* Efficient Electrocatalytic CO2 Reduction to C2+ Alcohols at Defect-Site-Rich Cu Surface. *Joule* **5**, 429-440 (2021).

9. Kastlunger, G., Heenen, H. H. & Govindarajan, N. Combining First-Principles Kinetics and Experimental Data to Establish Guidelines for Product Selectivity in Electrochemical CO2 Reduction. *ACS Catal.* **13**, 5062-5072 (2023).

10. Huang, Y., Handoko, A. D., Hirunsit, P. & Yeo, B. S. Electrochemical Reduction of CO2 Using Copper Single-Crystal Surfaces: Effects of CO* Coverage on the Selective Formation of Ethylene. *ACS Catal.* **7**, 1749-1756 (2017).

11. Li, J., *et al.* Constraining CO coverage on copper promotes high-efficiency ethylene electroproduction. *Nat. Catal.* **2**, 1124-1131 (2019).

12. Lum, Y. & Ager, J. W. Evidence for product-specific active sites on oxide-derived Cu catalysts for electrochemical CO2 reduction. *Nat. Catal.* **2**, 86-93 (2019).

13. García de Arquer, F. P., *et al.* CO2 electrolysis to multicarbon products at activities greater than 1 A cm-2. *Science* **367**, 661-666 (2020).

14. Dinh, C.-T., *et al.* CO2 electroreduction to ethylene via hydroxide-mediated copper catalysis at an abrupt interface. *Science* **360**, 783 (2018).

15. Zhong, M., *et al.* Accelerated discovery of CO2 electrocatalysts using active machine learning. *Nature* **581**, 178-183 (2020).

16. Wei, P., *et al.* Coverage-driven selectivity switch from ethylene to acetate in high-rate CO2/CO electrolysis. *Nat. Nanotechnol.* **18**, 299-306 (2023).

17. Ozden, A., *et al.* High-Rate and Efficient Ethylene Electrosynthesis Using a Catalyst/Promoter/Transport Layer. *ACS Energy Lett.* **5**, 2811-2818 (2020).

18. Tan, Y. C., Lee, K. B., Song, H. & Oh, J. Modulating Local CO2 Concentration as a General Strategy for Enhancing C−C Coupling in CO2 Electroreduction. *Joule* **4**, 1104-1120 (2020).

19. Ma, M., *et al.* Insights into the carbon balance for CO2 electroreduction on Cu using gas diffusion electrode reactor designs. *Energy Environ. Sci.* **13**, 977-985 (2020).

20. Huang, J. E., *et al.* CO2 electrolysis to multicarbon products in strong acid. *Science* **372**, 1074-1078 (2021).

21. She, X., *et al.* Tandem Electrodes for Carbon Dioxide Reduction into C2+ Products at Simultaneously High Production Efficiency and Rate. *Cell Rep. Phys. Sci.* **1**, 100051 (2020).

22. Zhang, T., *et al.* Highly selective and productive reduction of carbon dioxide to multicarbon products via in situ CO management using segmented tandem electrodes. *Nat. Catal.* **5**, 202-211 (2022).

23. Chen, C., *et al.* Cu-Ag Tandem Catalysts for High-Rate CO2 Electrolysis toward Multicarbons. *Joule* **4**, 1688-1699 (2020).

24. Hoang, T. T. H., *et al.* Nanoporous Copper-Silver Alloys by Additive-Controlled Electrodeposition for the Selective Electroreduction of CO2 to Ethylene and Ethanol. *J. Am. Chem. Soc.* **140**, 5791-5797 (2018).

25. Romero Cuellar, N. S., *et al.* Two-step electrochemical reduction of CO2 towards multi-carbon products at high current densities. *J. CO2 Util.* **36**, 263-275 (2020).

26. Ozden, A., *et al.* Cascade CO2 electroreduction enables efficient carbonate-free production of ethylene. *Joule* **5**, 706-719 (2021).

27. Wu, G., *et al.* Selective Electroreduction of CO2 to n-Propanol in Two-Step Tandem Catalytic System. *Adv. Energy Mater.* **12**, 2202054 (2022).
