# Supplementary material for: A CO2 electrolyzer tandem cell system for CO2-CO co-feed valorization in a Ni-N-C/Cu-catalyzed reaction cascade
Source: Nat Commun. 2023 Sep 14;14:5680. doi: 10.1038/s41467-023-41278-7 (PMC10502113; doi:10.1038/s41467-023-41278-7)
Supplement: Supplementary file 2 — Peer Review File [file 41467_2023_41278_MOESM2_ESM.pdf]

nature portfolio

Peer Review File

A CO2 Electrolyzer Tandem Cell System for CO2-CO co-feed valorization in a Ni-N-C/Cu-catalyzed Reaction Cascade

Open Access This file is licensed under a Creative Commons Attribution 4.0 International License, which permits use, sharing, adaptation, distribution and reproduction in any medium or format, as long as you give appropriate credit to the original author(s) and the source, provide a link to the Creative Commons license, and indicate if changes were made. In the cases where the authors are anonymous, such as is the case for the reports of anonymous peer reviewers, author attribution should be to 'Anonymous Referee' followed by a clear attribution to the source work. The images or other third party material in this file are included in the article's Creative Commons license, unless indicated otherwise in a credit line to the material. If material is not included in the article's Creative Commons license and your intended use is not permitted by statutory regulation or exceeds the permitted use, you will need to obtain permission directly from the copyright holder. To view a copy of this license, visit http://creativecommons.org/licenses/by/4.0/.

Editorial Note: This manuscript has been previously reviewed at another journal that is not operating a transparent peer review scheme. This document only contains reviewer comments and rebuttal letters for versions considered at *Nature Communications* . Mentions of the other journal have been redacted.

REVIEWERS' COMMENTS

Reviewer #1 (Remarks to the Author):

The authors have done a nice work to address my comments raised in the last round for another [Redacted] journal. Considering the importance of this topic and advance of knowledge gained in the work, I believe it is now suitable for publication in Nature Communications.

Reviewer #2 (Remarks to the Author):

After receiving initial feedback and comments from reviewers, the authors have made significant efforts to address the concerns raised regarding the novelty and suitability of their manuscript for [Redacted]. Reviewers noted the lack of new mechanistic insights, novel materials, and cell designs, which potentially limited the manuscript's appropriateness for publication in [Redacted].

In response to these critiques, the authors have done a good job in revising their work and providing detailed new information. They have expanded the context of their study, placing the findings within a broader perspective for comparison with existing research in the field. This indicates the manuscript's potential for publication in Nature Communications.

The authors' efforts to provide additional context and demonstrate the significance of their work in a broader context indicate a successful response to the reviewers' comments. By doing so, they have strengthened the manuscript's scientific contribution and relevance, making it more suitable for publication in Nature Communications. Overall, the authors' diligence in addressing the reviewers' feedback and enhancing the manuscript's content has significantly improved its suitability for publication, now positioning it well for consideration in Nature Communications.
